# Screening of Antagonistic *Trichoderma* Strains to Enhance Soybean Growth

**DOI:** 10.3390/jof11020159

**Published:** 2025-02-19

**Authors:** Na Yu, Yijia Gao, Feng Chang, Wenting Liu, Changhong Guo, Hongsheng Cai

**Affiliations:** Key Laboratory of Molecular Cytogenetics and Genetic Breeding of Heilongjiang Province, College of Life Science and Technology, Harbin Normal University, Harbin 150025, China

**Keywords:** *Trichoderma*, *Fusarium*, rhizosphere, plant growth promotion, biological control

## Abstract

This study investigates the isolation and screening of *Trichoderma* strains that exhibit antagonistic properties against soybean root-infecting *Fusarium* species, particularly *F. oxysporum*. From soybean rhizosphere soil, 37 antagonistic *Trichoderma* strains were identified using the plate confrontation method, demonstrating inhibitory effects ranging from 47.57% to 72.86% against *F. oxysporum*. Strain 235T4 exhibited the highest inhibition rate at 72.86%. Molecular identification confirmed that the strains belonged to eight species within the *Trichoderma* genus, with notable strains promoting soybean growth in greenhouse tests. In pot experiments, the application of *Trichoderma* significantly reduced the disease index of soybean plants inoculated with *F. oxysporum*, particularly with strain 223H16, which achieved an 83.78% control efficiency. Field applications further indicated enhanced soybean growth metrics, including increased pod numbers and plant height, when treated with specific *Trichoderma* strains. Additionally, *Trichoderma* application enriched the fungal diversity in the soybean rhizosphere, resulting in a significant reduction of *Fusarium* populations by approximately 50%. This study highlights the potential of *Trichoderma* species as biological control agents to enhance soybean health and productivity while improving soil fungal diversity.

## 1. Introduction

The soybean plant (*Glycine max*) plays a crucial role in global agriculture, serving as a major source of protein and vegetable oil. However, soybean cultivation faces numerous challenges, including soil-borne diseases caused by various pathogens that can significantly impact crop yield and quality [1]. In recent years, there has been growing interest in exploring sustainable and environmentally friendly alternatives to chemical-based disease control methods [2]. One promising approach involves the use of beneficial microorganisms, such as *Trichoderma* spp., to enhance plant growth and suppress soil-borne pathogens.

*Fusarium* diseases in soybean are a significant concern in agricultural production [3,4]. *Fusarium* is a genus of fungi that can cause various diseases in soybean plants, leading to reduced yield and quality. Some common *Fusarium* diseases affecting soybeans include *Fusarium* wilt caused by *F. oxysporum*, sudden death syndrome (SDS) caused by *F. virguliforme*, and several species causing seed and seedling diseases [5]. Soybean root rot, caused by the fungal pathogen *Fusarium*, is a significant concern in soybean production worldwide [6,7,8]. *Fusarium* species are commonly found in soil and can infect soybean plants at various stages of growth, leading to devastating consequences for crop yield and quality. This destructive disease is characterized by the decay and discoloration of soybean roots, which can eventually result in stunted growth, wilting, and even plant death. *Fusarium* root rot not only directly affects the health of soybean plants but also weakens their overall vigor, making them more susceptible to other stressors, such as drought and nutrient deficiencies [9]. Managing soybean root rot caused by *Fusarium* is a pressing challenge for both farmers and researchers, as several *Fusarium* species can cause disease. This requires an integrated approach that involves cultural practices, genetic resistance, and the use of effective control measures to mitigate the impact on soybean production.

The use of fungicide-treated seeds is considered an effective management strategy to prevent *Fusarium* infections in commercial soybean fields. However, many soybean farmers often choose not to adopt this practice due to the perceived low cost-benefit ratio, especially considering that disease incidence is influenced by weather conditions, with a higher likelihood of occurrence in cold and wet environments. The uncertainty of disease occurrence and the associated costs of applying fungicides contribute to the hesitation among farmers to adopt this preventive measure.

*Trichoderma* spp. are ubiquitous filamentous fungi present in diverse soil ecosystems. They have been widely studied for their antagonistic properties against a wide range of plant pathogens [10,11]. These beneficial fungi exhibit various mechanisms, including antibiosis, mycoparasitism, and induction of plant defense responses, which contribute to their biocontrol potential. Moreover, *Trichoderma* spp. are known for their ability to promote plant growth through mechanisms such as nutrient solubilization, phytohormone production, and improved nutrient uptake [12].

The rhizosphere, the soil region surrounding plant roots, represents a dynamic niche where plant–microbe interactions occur [13]. It is rich in organic matter and provides a favorable environment for the growth and activity of beneficial microorganisms. The rhizosphere of soybean plants has been shown to harbor a diverse microbial community, including *Trichoderma* spp., which can positively influence plant health and growth. The isolation and identification of *Trichoderma* spp. has gained considerable attention due to its potential for developing sustainable strategies for disease management. By isolating specific strains of *Trichoderma* spp., researchers aim to identify those with superior biocontrol and plant growth-promoting abilities. These selected strains can then be further evaluated and optimized for field application.

This study focused on the screening of antagonistic *Trichoderma* spp. from the soybean rhizosphere and tested their beneficial properties on soybean plants under both greenhouse and field conditions, aiming to explore their roles in promoting plant growth and enhancing soil microbial communities. This research will not only deepen our understanding of the multiple beneficial properties of *Trichoderma* spp. exhibited on plants but will also contribute to the development of sustainable and effective disease management strategies for soybean cultivation, with the ultimate goal of improving crop productivity and ensuring food security.

## 2. Materials and Methods

### 2.1. Isolation and Screening of Trichoderma from Soybean Rhizosphere

Rhizosphere soil was collected from healthy soybean plants in the main soybean production area of Heilongjiang Province. To prepare the initial soil suspension, 5 g of soybean rhizosphere soil was weighed and mixed with 45 g of sterile water, resulting in a 1:10 dilution. The mixture was placed on a shaker and agitated at a speed of 150 rpm for 20 min. The soil suspension was then gradually diluted using a gradient dilution method to obtain suspensions with concentrations of 10^−2^ and 10^−3^. Using a micropipette, 150 μL of each soil suspension was transferred onto V8 agar medium and evenly spread with a sterilized spreading rod, with two replicates for each concentration. The plates were incubated in the dark at 28 °C for 3 days. After observing the formation of fungal colonies, the number of colonies was recorded. Single, well-grown colonies were selected and transferred to PDA plates for further cultivation for 5 days to obtain purified fungal isolates.

The antagonistic effects of the isolates on *F. oxysporum* (stored in this lab) were screened using the plate confrontation method. Using a 5 mm puncher, holes were made on the PDA plates, and on both sides of each plate, 2 cm away from the edge, *F. oxysporum* and the test isolate were inoculated. A 5 mm *F. oxysporum* mycelial plug was used as a control at the center of the PDA plate. Each group was replicated twice, and the experiment was repeated three times. The plates were incubated in a constant temperature incubator at 28 °C in the dark for 5 days, and the growth of the colonies was observed. The isolates that significantly inhibited the growth of *F. oxysporum* were considered antagonistic strains. The colony radius of *F. oxysporum* was measured.

### 2.2. Effect of Trichoderma on Soybean Seed Germination

*Trichoderma* strains were cultured on PDA medium for 7 days to allow sufficient mycelial growth. A suitable amount of sterile water was drawn and placed in a Petri dish. Using a sterilized surgical knife, the mycelium was gently scraped and filtered through eight layers of sterile gauze into a conical flask. The Petri dish was rinsed repeatedly with a small amount of sterile water to obtain a certain concentration of spore suspension. A dropper was used to draw a small amount of well-shaken and mixed spore solution onto a hemocytometer. After a brief period of settling, the spores were counted under a microscope. Finally, the spore suspension concentration was adjusted to 1 × 10^8^ spores/mL.

In a laminar flow hood, soybean seeds were soaked in 75% ethanol for 5 min and continuously shaken for surface sterilization. They were then rinsed with sterile water six times. Six soybean seeds were selected and placed in a culture dish with a layer of moist filter paper. Each seed was treated with 200 μL of the 1 × 10^8^ spores/mL spore suspension, and an equal amount of sterile distilled water was applied as a control. The experiment was performed in triplicate. The dishes were incubated in the dark for 5 days, and the germination and growth of the seeds were observed. The seed growth rate was calculated.

### 2.3. Molecular Identification of Trichoderma

After culturing the tested strain on PDA medium plates for 7 days, the mycelium was scraped and frozen in liquid nitrogen, then ground into powder with a mortar and pestle. The modified CTAB method was used to extract the strain DNA. Two pairs of primers, ITS1 (TCCGTAGGTGAACCTGCGG) and ITS4 (TCCTCCGCTTATTGATATGC) [14], and TEF71F (CAAAATGGGTAAGGAGGASAAGAC) and TEF997R (CAGTACCGGCRGCRATRATSAG) [15], were used for amplification. The PCR reaction system for both ITS and TEF primers was 25 μL, including 1.5 μL of DNA template, 1 μL of each forward and reverse primer, 12.5 μL of PCR Mix (purchased from Shanghai Sangon Biotech Co., Ltd., Shanghai, China), and 9 μL of sterile water. The PCR amplification conditions for ITS were as follows: initial denaturation at 95 °C for 2 min, denaturation at 95 °C for 30 s, annealing at 60 °C for 30 s, extension at 72 °C for 45 s, repeated for 30 cycles, and final extension at 72 °C for 6 min. The PCR amplification conditions for TEF were as follows: initial denaturation at 95 °C for 6 min, denaturation at 95 °C for 1 min, annealing at 58 °C for 30 s, extension at 72 °C for 1 min, repeated for 30 cycles, and final extension at 72 °C for 5 min. A small amount of PCR product was subjected to 1% agarose gel electrophoresis, and once PCR amplification bands were observed, the remaining PCR products were sent to Shanghai Sangon Biotech Co., Ltd. for sequencing.

The sequencing results were submitted to NCBI (https://www.ncbi.nlm.nih.gov/) for nucleotide sequence alignment (Supplementary Table S1), accessed on 20 February 2024. The ITS and TEF sequences with high homology were downloaded, and MEGA11 software was used to construct phylogenetic trees using the maximum likelihood method [16]. The classification status of the *Trichoderma* strain was further determined based on morphology and molecular phylogenetic tree analysis.

### 2.4. Effects of Trichoderma spp. on Soybean Growth in Greenhouse Conditions

To prepare the substrate, a mixture of corn flour, wheat bran, and sterile water was combined in a ratio of 1:1:2. This mixture was thoroughly stirred and sterilized at 121 °C for 20 min. A spore suspension of *Trichoderma*, with a concentration of 1 × 10^8^ spores, was then mixed with the substrate at a ratio of 1:15 to create the *Trichoderma* agent. The mixture was incubated in the dark at a constant temperature of 28 °C for 3 days, with daily thorough mixing to ensure uniformity. For the control group, an equivalent amount of distilled water was added to the substrate.

Greenhouse pot experiments were conducted to evaluate the growth-promoting effects of *Trichoderma* strains on soybean plants, utilizing the soybean cultivar ‘Xiaojinhuang’ (stored in this lab) as the test crop. A total of 5 pots were used, each containing approximately 700 g of sterilized vermiculite mixed with the *Trichoderma* agent at a ratio of 25:1. Six seeds were sown in each pot. To maintain adequate moisture levels in the vermiculite, each pot received 500 mL of nutrient solution weekly. The pots were arranged in the greenhouse under alternating light and dark conditions. In the control group, distilled water was used in place of the *Trichoderma* agent. Each treatment was replicated three times.

After a growth period of 30 days, the soybean plants were carefully removed from the pots, and residual vermiculite was rinsed from the roots using water. The biomass of the plants was measured with a ruler and a precision electronic balance, assessing parameters such as plant height, root length, fresh weight, and dry weight. Subsequently, the plants were wrapped and placed in a constant temperature drying oven at 80 °C until a constant weight was reached, at which point their dry weight was recorded.

### 2.5. Biocontrol of Trichoderma on Soybean Root Rot Caused by F. oxysporum Under Pot Experiment

To investigate the biocontrol efficacy of *Trichoderma* against root rot in soybean caused by *F. oxysporum*, a pot experiment was conducted. A mixture of sterilized vermiculite, *Trichoderma* agent, and *F. oxysporum* agent was prepared in a ratio of 25:1:1 and placed into flower pots. Six soybean seeds were planted in each pot, and the pots were maintained in a greenhouse under alternating light and dark conditions. The plants were irrigated weekly with a nutrient solution.

A control group was established, consisting of pots inoculated only with the *F. oxysporum* agent. Each treatment group was replicated three times to ensure statistical validity. After 30 days, the soybean plants were removed from the pots, and the residual vermiculite was rinsed from the roots using water. Surface moisture was absorbed using filter paper, and the incidence and severity of *F. oxysporum* infection on the soybean roots were recorded based on the following criteria:

Level 0: Primary and lateral roots are healthy with no disease symptoms;

Level 1: Slight brown lesions on the primary and lateral roots, with normal plant growth;

Level 2: Darkening of brown lesions on the primary roots, with normal plant growth;

Level 3: Brown lesions on the primary roots turning black, with fewer lateral roots;

Level 4: Most of the primary roots turn black with no obvious lateral roots and slow plant growth;

Level 5: Lesions surround the entire root system, complete rotting of the roots, plants cannot grow normally and may wither and die.

The following metrics were calculated to assess the disease impact and control efficacy:

$$\mathrm{Disease}\;\mathrm{incidence}\;(\mathrm{DI})=\left(\frac{\mathrm{Number}\;\mathrm{of}\;\mathrm{diseased}\;\mathrm{plants}}{\mathrm{Total}\;\mathrm{number}\;\mathrm{of}\;\mathrm{plants}}\right)\times 100\%$$

Disease severity index (DSI):

$$\mathrm{DSI}=\frac{\Sigma (\mathrm{Number}\;\mathrm{of}\;\mathrm{plants}\;\mathrm{at}\;\mathrm{each}\;\mathrm{level}\times \mathrm{Relative}\;\mathrm{value}\;\mathrm{of}\;\mathrm{the}\;\mathrm{level})}{\mathrm{Total}\;\mathrm{number}\;\mathrm{of}\;\mathrm{plants}\;\mathrm{surveyed}\;\times \;\mathrm{Highest}\;\mathrm{value}\;\mathrm{of}\;\mathrm{the}\;\mathrm{level}}\times 100\%$$

Relative control effect (RCE):

$$\mathrm{RCE}=\frac{\mathrm{DSI}\;\mathrm{of}\;\mathrm{the}\;\mathrm{control}\;\mathrm{group}-\mathrm{DSI}\;\mathrm{of}\;\mathrm{the}\;\mathrm{treatment}\;\mathrm{group}}{\mathrm{DSI}\;\mathrm{of}\;\mathrm{the}\;\mathrm{control}\;\mathrm{group}}\times 100\%$$

### 2.6. Evaluation of Trichoderma on Soybean Production in the Field

The field experiment was conducted at Harbin Normal University, located at approximately latitude 45.75° N and longitude 126.65° E. The experiment took place over a duration from 1 May to 15 October 2023, during which temperature and rainfall data were recorded, with average temperatures ranging from 12 °C to 25 °C and average rainfall measuring 586.4 mm.

The soybean cultivars tested were Mengdou and Xingnong (stored in this lab), and the biocontrol agent utilized was *Trichoderma,* specifically strains 223H16, 452B7, 561A7, 611A17, and 625J11, known for their significant antagonistic and growth-promoting effects. Each planting area for the soybean varieties was designed with a ridge length of 1 m, a ridge width of 0.3 m, and a spacing of 0.3 m between plants. A total of 100 g of *Trichoderma* substrate, cultured for three days, was evenly distributed in the furrow of each ridge, with 30 soybean seeds sown per ridge. The blank control group received no *Trichoderma* substrate.

The two soybean planting areas measured 3 m in length and 7 m in width, containing 11 treatment groups, each receiving different *Trichoderma* treatments. Regular weeding was conducted throughout the planting period, and no fertilizers or pesticides were applied.

At the first trifoliate stage, 21 days after planting, ten soybean seedlings were randomly selected from each treatment group to measure parameters such as plant height, dry weight, and fresh weight. At the podding stage, another ten soybean plants were randomly selected from each treatment group to assess their pod characteristics. Upon maturity, all soybean plants were harvested, and data on plant height and yield were recorded.

### 2.7. Soil DNA Extraction and High-Throughput Sequencing

During the harvest of soybeans at maturity, root zone soil samples from each treatment group and the control group were randomly collected. Three samples were collected from each treatment group and placed in sterile sample bags. The magnetic bead method was used to extract DNA from soil samples, and the quality of the DNA was assessed using 1% agarose gel electrophoresis. Primers for the fungal internal transcribed spacer (ITS) region, specifically ITS3R: GCATCGATGAAGAACGCAGC and ITS4F: TCCTCCGCTTATTGATATGC, were selected for PCR amplification of the soil fungal ITS2 region. PCR amplification was performed using TransStart Fastpfu DNA Polymerase (Shanghai Sangon Biotech Co., Ltd., Shanghai, China) with a reaction system of 20 μL, comprising 5× FastPfu Buffer 4 μL, 2.5 mM dNTPs 2 μL, Forward Primer (5 μM) 0.8 μL, Reverse Primer (5 μM) 0.8 μL, FastPfu Polymerase 0.4 μL, Template DNA 30 ng, and ddH_2_O to a final volume of 20 μL. The PCR reaction parameters were set as follows: 98 °C for 1 min for pre-denaturation; 98 °C for 10 s for denaturation; 50 °C for 30 s for annealing; 72 °C for 60 s for extension, repeated for 30 cycles, followed by a final extension at 72 °C for 10 min. The amplification products were detected using 2% agarose gel electrophoresis, and the target bands were recovered. The PCR amplification products were purified using Agencourt AMPure XP magnetic beads and dissolved in an Elution Buffer. The fragment range and concentration of the library were assessed using the Agilent 2100 Bioanalyzer (Headquarters, Santa Clara, CA, USA), and a Meta rDNA Amplicon library was constructed. Sequencing and analysis were conducted by the Wuhan BGI Gene Technology Service Co., Ltd. through the Illumina MiSeq high-throughput sequencing platform.

### 2.8. Data Analysis

The experiments were conducted in triplicate, adhering to a completely randomized design to ensure reliability and minimize bias. Data analysis was performed using SPSS 20 statistical software (IBM Corp., Armonk, NY, USA). For each experiment, the means of the collected data were calculated and analyzed using Duncan’s multiple range test to determine significant differences among treatment groups. The significance level was established at *p* ≤ 0.05, allowing for the identification of statistically significant variations in the results. For the analysis of sequencing data, data filtering and subsequent analysis involved several key steps to process the raw sequences obtained from sequencing. Initially, the sequences were processed using cutadapt v2.6 software to generate Clean Data. This involved trimming reads matching the primers to eliminate primer and adapter contamination, resulting in fragments of the target region. A window length of 25 bp was employed to trim the ends of reads that had an average quality value below 20, and any reads with final lengths less than 75% of the original length were discarded. Additionally, reads containing ‘N’ were excluded, as were those with consecutive low complexity of 10 ATCG, leading to the final Clean Data for further analysis. For tag linking and OTU clustering, high-quality clean reads from paired-end sequencing were assembled into single sequences using FLASH software (Fast Length Adjustment of Short reads, v1.2.11), with assembly conditions set for a minimum overlap length of 15 bp and a permissible mismatch rate of 0.1 in the overlap region, producing high-variable region Tags. Clustering was then conducted using USEARCH v12 at a 97% similarity threshold to derive representative sequences of OTUs. Chimeric sequences in the OTU representatives were removed using UCHIME (v4.2.40), and the Usearch global method mapped all Tags back to the OTU representative sequences, creating an abundance table of OTUs for each sample. Finally, species annotation was carried out using the RDP classifier (1.9.1) with the Unite database through blastn comparison, applying a confidence threshold of 0.6 while also excluding OTUs without annotation results and those not classified as fungi.

## 3. Results

### 3.1. Isolating and Screening Trichoderma Strains Antagonistic to Soybean Root-Infecting Fusarium Species

From the collected health soybean rhizosphere soil, pure and separated fungi were obtained using the dilution spread method. The most aggressive strain of *F. oxysporum* isolated from soybean root rot disease in our previous study [7] was selected as the target fungus, and 37 strains of antagonistic *Trichoderma* exhibiting significant inhibitory effects were screened using the plate confrontation method (Supplementary Figure S1). The inhibition rates of the 37 antagonistic strains against soybean *F. oxysporum* were statistically analyzed on agar plates, and the results showed that the inhibitory effects ranged from 47.57% to 72.86% (Table 1). Among them, strain 235T4 showed the most significant inhibition effect against *F. oxysporum*, with an inhibition rate of 72.86%. The strains 632H1 and 231K4 also exhibited relatively high inhibition rates against *F. oxysporum*, with rates of 67.87% and 67.75%, respectively. The strain 234N4 had a lower inhibition rate of 47.57%. The antagonistic strains exhibited radial growth on the agar plates, with rapid hyphal growth and the ability to quickly occupy the growth space. *F. oxysporum* was unable to grow normally due to inhibition by the antagonistic strains, and there were even instances where the colonies of *F. oxysporum* were covered by the antagonistic Trichoderma.

We also tested the inhibitory effects of 37 strains of *Trichoderma* fungi on four other soybean root rot pathogens, e.g., *F. solani*, *F. equiseti*, *F. proliferatum*, and *F. graminearum* (stored in this lab). The results show that the 37 antagonistic strains have varying degrees of inhibitory effects on the four *Fusarium* species (Supplementary Table S1).

### 3.2. Molecular Identification of Trichoderma Species

Based on the morphological observations and molecular identification results, it was ultimately determined that the 37 antagonistic strains belong to eight species within the *Trichoderma* genus. Strain 121A15 clusters with *T. viride* in the same branch; Strain 225J10 clusters with *T. velutinum* in the same branch; Strain 234N4 clusters with *T. navalis* in the same branch; Strain 342G10 clusters with *T. gamsii* in the same branch; Strains 244S9, 321F10, 324I4, and 324I5 cluster with *T. hamatum* in the same branch. Strains 134D16, 141F9, 231K16, 235T4, 243R6, 333C17, 551A6, 561A7, 625J11, and 651A8 are grouped with *T. koningiopsis* in the same branch. Strains 215E14, 223H3, 223H16, 231K4, 311A2, 333C1, 343I8, 461C1, 415E2, 452B7, 553C1, 563C3, 563C5, 611A17, 611A18, 612B8, and 623H1 belong to the same branch as *T. harzianum*. Strains 552B7 and 614D1 cluster with *T. atroviride* in the same branch. The characteristics of colony and spore structure, as well as the phylogenetic trees based on ITS and TEF genes, are listed in Supplementary Figures S2 and S3.

### 3.3. Effects of Trichoderma Species on Soybean Plant Growth in Greenhouse

**Figure 1:**
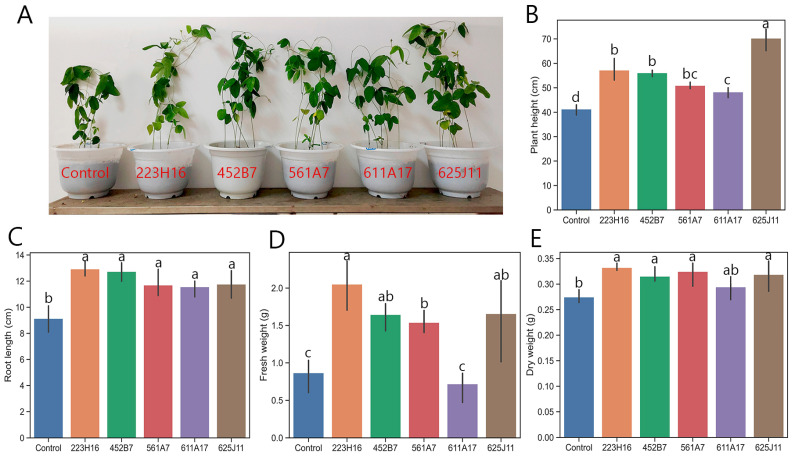
The effects of *Trichoderma* treatment on the phenotype and biomass of potted soybean plants. (**A**) Phenotype of soybeans after Trichoderma treatment. (**B**) plant height (cm). (**C**) root length (cm). (**D**) fresh weight (g). (**E**) dry weight (g). Data are present as means ± standard deviation; Different letters in the same column indicate significant differences between different treatments (*p* < 0.05).

The moistened filter paper plate method was used to assess the growth-promoting effects of 37 *Trichoderma* strains with good antagonistic capabilities on soybean seeds. The results revealed that five *Trichoderma* strains, namely, 223H16, 452B7, 561A7, 611A17, and 625J11, exhibited significant abilities to promote soybean germination and growth. The plants treated with different *Trichoderma* agents exhibited varying degrees of growth-promoting effects (Figure 1). In terms of plant height, soybean plants treated with the 223H16, 452B7, 561A7, 611A17, and 625J11 strains exhibited significant increases in height compared to the control group (*p* < 0.05). Among them, the 625J11 strain showed the most significant growth-promoting effect, with an increase of 58.73% compared to the control. The other strains, including 223H16, 452B7, 561A7, and 611A17, resulted in height increases of 33.10%, 32.31%, 21.86%, and 14.62%, respectively, compared to the control. The 452B7 strain showed the most significant increase in root length, with a 21.52% improvement compared to the control. Additionally, the 223H16, 561A7, 611A17, and 625J11 strains also significantly enhanced root elongation in soybean, with increases of 20.58%, 17.76%, 15.79%, and 18.61%, respectively, compared to the control (*p* < 0.05). In terms of dry weight, soybean plants treated with the 223H16 strain showed the highest dry weight, with a 21.00% increase compared to the control (*p* < 0.05). There were no significant differences in dry weight between the 611A17 strain treatment and the control group, while the rest of the strain treatments showed significant increases compared to the control (*p* < 0.05). Regarding the fresh weight of soybean plants, except for the 611A17 strain, which showed no significant difference compared to the control, the other four *Trichoderma* strains significantly increased the fresh weight of soybean plants (*p* < 0.05). Among them, the 223H6 strain showed the most significant difference compared to the control, with a remarkable increase of 70.77%.

### 3.4. Potting Experiments to Determine the Control Effect of Trichoderma on Soybean Root Rot Disease

In greenhouse potting experiments, significant differences in the disease index of soybean plants were observed after simultaneous inoculation with *Trichoderma* and *F. oxysporum* for 30 days (Table 2). Soybean plants inoculated solely with *F. oxysporum* showed severe disease symptoms, with a disease incidence rate of 83.33% and a disease index of 77.08%. Compared to the control group, soybean plants treated with different *Trichodermas* showed a significant decrease in disease symptoms, with disease indices ranging from 12.50% to 33.33% and relative control efficiencies ranging from 56.76% to 83.78%. Among them, *Trichoderma* 223H16 showed the best control effect, reducing the disease index to 12.50% and achieving a relative control efficiency of 83.78%. The *Trichoderma* 452B7 and 561A7 had the second-best control effect, with a disease index of 16.66% and a relative control efficiency of 78.39%. The *Trichoderma* 611A17 had the relatively weakest control effect on *F. oxysporum*, with a disease index of 33.33% and a relative control efficiency of 56.76% (*p* < 0.05).

### 3.5. Field Application Research of Selected Beneficial Trichoderma spp. for Soybean

At the first trifoliate stage, there were differences in the biomass improvement of soybean cv. Mung bean and Xingnong were among the five *Trichoderma* strains compared to the control group (Table 3). In soybean cv. Mung bean, the tallest plant increase was most significant with the treatment of the 452B7 strain, which showed a 19.35% increase. There were no significant differences in fresh weight among the different *Trichoderma* treatments compared to the control, while the 223H16 strain showed the most significant increase in dry weight at 6.03%. There were no significant differences in plant height, fresh weight, and dry weight between the soybean cv. Mung bean treated with 561A7, 611A17, and 625J11 strains, and the control group. The 625J11 strain had a significant effect on increasing plant height, fresh weight, and dry weight in soybean cv. Xingnong, with increases of 18.08%, 24.96%, and 6.78%, respectively. Similarly, the 611A17 strain also had a noticeable effect on increasing plant height, fresh weight, and dry weight in soybean cv. Xingnong, with increases of 14.19%, 22.81%, and 5.73%, respectively. There were no significant differences in plant height, fresh weight, and dry weight between the 452B7 and 561A7 strains compared to the control group in soybean cv. Xingnong.

The statistical analysis of soybean data at the mature harvest stage indicates that compared to the control group without *Trichoderma* application, the strains 223H16, 452B7, 561A7, 611A17, and 625J11 significantly increased the number of pods in soybean cv. Mung bean (Table 4). They showed respective improvements of 61.82%, 38.18%, 56.36%, 21.82%, and 30.91% compared to the control. The strains 223H16 and 561A7 promoted plant height in soybeans, with increases of 27.65% and 18.24%, respectively. Among the five Trichoderma strains, 452B7, 561A7, and 611A17 significantly increased the hundred-grain weight of soybean cv. Mung bean, with increases of 4.83%, 9.76%, and 5.48%, respectively. In soybean cv. Xingnong, the application of 223H16, 452B7, 561A7, 611A17, and 625J11 strains significantly increased the number of pods, with increases of 71.74%, 69.57%, 65.22%, 67.39%, and 41.30%, respectively. The strains 223H16, 561A7, and 625J11 significantly increased the plant height of soybean cv. Xingnong, with increases of 22.87%, 22.87%, and 9.04%, respectively. The strains 452B7, 611A17, and 625J11 significantly increased the hundred-grain weight of soybean cv. Xingnong, with improvements of 8.39%, 3.31%, and 10.92%, respectively, compared to the control group.

### 3.6. The Impact of Applying Trichoderma Agents on the Fungal Community Structure in the Soil

**Figure 2:**
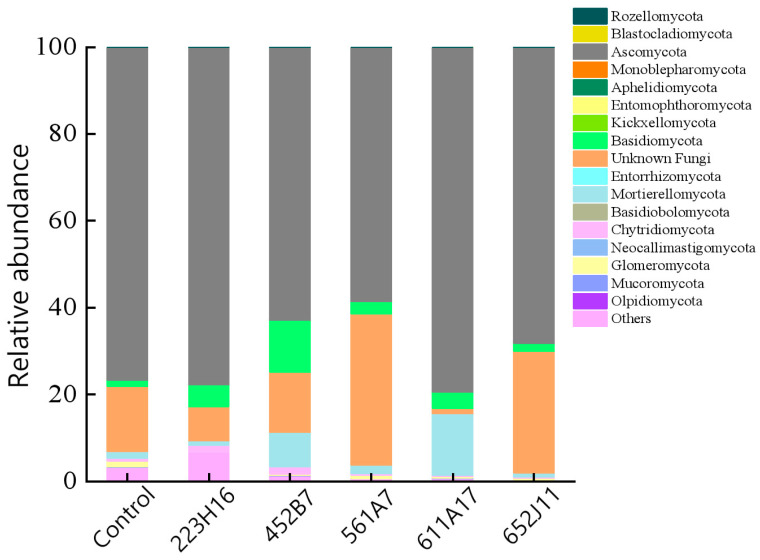
Differences in fungal community compositions among *Trichoderma* treatments. This column chart displays the relative abundances of species at the phylum level.

The composition of soil fungi in the rhizosphere of soybeans treated with different *Trichoderma* fungi varies. The relative abundance of fungal communities at the phylum level for different treatment groups is shown in Figure 2. A total of 16 fungal phyla were identified from the sequencing results, mainly including Olpidiomycota, Mucoromycota, Glomeromycota, Neocallimastigomycota, Chytridiomycota, Basidiobolomycota, Mortierellomycota, Entorrhizomycota, Basidiomycota, Kickxellomycota, Entomophthoromycota, Aphelidiomycota, Monoblepharomycota, Ascomycota, Blastocladiomycota, Rozellomycota, and unknown fungi. After treatment with *Trichoderma*, the relative abundances of Basidiobolomycota and Monoblepharomycota increased. No regular changes were observed for the other phyla.

The analysis results of fungal diversity in the fields are shown in Supplementary Table S1. Taking the Shannon and Chao indices as examples, the results presented in Table 5 indicate the fungal diversity indices, specifically the Chao and Shannon indices, across different treatments. The control group exhibited a Chao index of 512.01 ± 55.52 and a Shannon index of 2.32 ± 0.12, suggesting relatively lower fungal diversity. In contrast, treatment 611A17 showed the highest Chao index at 705.9 ± 53.99 and the highest Shannon index at 3.48 ± 0.54, indicating significantly greater species richness and diversity. The experimental group 452B7 also demonstrated notable diversity, with a Chao index of 674.61 ± 50.89 and a Shannon index of 3.29 ± 0.43. Other treatments, such as 223H16 and 561A7, had intermediate values, with 223H16 showing a Chao index of 638.25 ± 93.5 and a Shannon index of 2.77 ± 0.32, while 561A7 recorded a Chao index of 625.58 ± 86.92 and a Shannon index of 2.84 ± 0.34. The significant differences, as indicated by different letters, highlight the varying effects of treatments on fungal diversity. Overall, the findings suggest that the colonization of Trichoderma in soybean soil enhances species richness in the soybean rhizosphere. Additionally, Good’s coverage index indicates that the sequencing depth was sufficient for this experiment.

**Figure 3:**
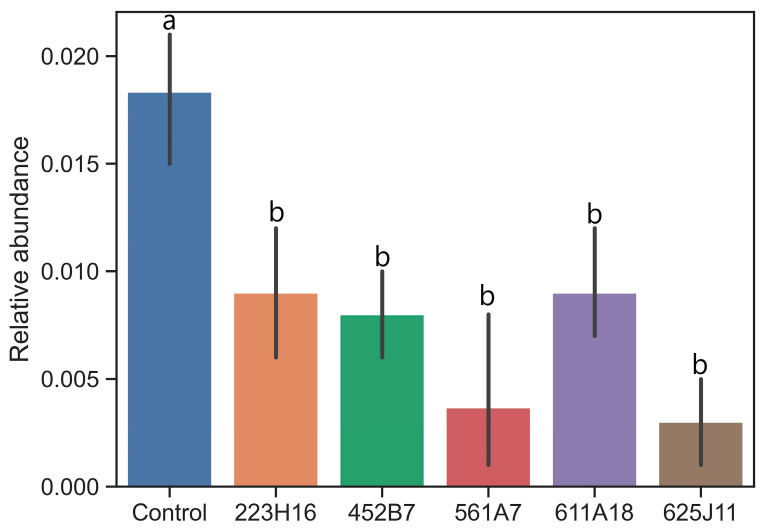
Relative abundance of *Fusarium* species in soybean rhizosphere soil in the field. Data are present as means ± standard deviation; Different letters in the same column indicate significant differences between different treatments (*p* < 0.05).

Due to the strong antagonistic effects of *Trichoderma* on various *Fusarium* species, we aimed to assess the distribution of *Fusarium* in the soil after applying *Trichoderma* in the field. The results obtained from ITS sequencing, illustrated in Figure 3, indicate a significant decreasing trend in the *Fusarium* genus in the soybean rhizosphere soil compared to the control group following the application of *Trichoderma*. Quantitative analysis revealed that *Fusarium* populations decreased by approximately 50% in treated soils. This reduction was correlated with an increase in beneficial fungi, such as mycorrhizal species, indicating a potential synergistic effect of *Trichoderma* on overall soil health.

## 4. Discussion

The incidence of soybean root rot caused by *Fusarium* in Northeast China has become more frequent, as observed in our previous large-scale survey [7]. With no effective disease-resistant varieties available and prevention being difficult, there is an urgent need for more environmentally friendly biological control methods. Indigenous microbial resources have proven to be effective biological control agents due to their good adaptability and stable performance [17]. These microorganisms are naturally occurring in specific environments, which allows them to thrive under local conditions and interact synergistically with native flora and fauna. Their ability to establish themselves in diverse ecosystems enhances their effectiveness in suppressing plant pathogens and promoting plant health. In this study, we aimed to screen *Trichoderma* spp. strains isolated from the rhizosphere of soybean plants and evaluate their application for soybean growth promotion and control of Fusarium root rot disease.

*Trichoderma* spp. have been confirmed to possess the ability to enhance nutrient supply, suppress plant pathogens, and boost plant defenses [18]. You et al. found that *Trichoderma koningiopsis* T-51 not only inhibits gray mold and *Fusarium* activity but also promotes the growth of *Arabidopsis* seedlings through its volatile organic compounds (VOCs), significantly increasing seedling size and weight [19]. Liu et al. applied the African *Trichoderma afroharzianum* T52 as a microbial fertilizer to lilac, and the results showed that TafrT52 promoted the shedding of diseased leaves and the production of new leaves while enhancing catalase (CAT) activity, thereby improving the plant’s resistance to diseases [20]. Nuangmek et al. discovered that the application of *T. phayaoense* could increase the height of melon seedlings as well as the dry weight of both the aerial parts and roots, effectively improving plant development. The soluble sugar content in melon fruits also significantly increased, enhancing the fruit’s quality [21]. Phoka et al. demonstrated that the VOCs from *Trichoderma asperelloides* PSU-P1 promoted the growth of *Arabidopsis*, with treated plants showing increased fresh weight, root length, and chlorophyll content compared to the control. The VOCs-treated *Arabidopsis* exhibited higher activity of defense-related enzymes (peroxidase, POD) than the control group [22]. The results of our screening process revealed a diverse population of *Trichoderma* spp. strains in the soybean rhizosphere, including *T. harzianum*, *T. virens*, *T. atroviride*, and *T. asperellum*, among others. These species have exhibited antagonistic activity against several soybean root rot pathogens, such as *Fusarium* spp. Five of these strains also showed plant growth promotion, i.e., plant height, shoot, and root biomass under the greenhouse experiments condition. Although all five strains demonstrated plant growth-promoting ability, they differ in some ways; for example, 611A17 showed no difference compared to the control in terms of fresh weight and dry weight. This result suggests different promoting mechanisms are involved. This result is consistent with previous reports [23,24]. These findings indicate that the selected *Trichoderma* spp. strains positively impact soybean growth promotion.

Due to the complexity and variability of field conditions, as well as the instability of the external environment, biological control agents can effectively manage soybean root rot and promote plant growth under potting conditions, but the same effects may not be observed in the field. The effectiveness of microbial agents in the field largely depends on their ability to colonize and proliferate at the plant root zone [25]. The colonization of microbial agents can be influenced not only by their own colonization characteristics but also by various environmental factors such as temperature, humidity, light, and the nutritional status of the plants, which in turn affects the efficacy of these agents in controlling plant diseases [26]. Bandeira et al. evaluated the impact of five species of *Trichoderma* inoculated on the leaves and roots of eucalyptus on plant growth. The results indicated that root inoculation with *Trichoderma* contributed to nutrient absorption and promoted eucalyptus growth, demonstrating good colonization characteristics [27]. In this study, the percentage of *Trichoderma* at the genus level in the treatment groups 452B7, 611A17, and 625J11 applied in soybean planting areas was significantly higher than in the control group, suggesting that the increase in relative abundance of these three *Trichoderma* species may be due to the applied *Trichoderma*, which also confirms the good colonization performance of biocontrol *Trichoderma* in soybean rhizosphere soil.

The dynamics of microbes and microbe interactions in plant root rhizospheres have recently been recognized as important features of the phyllosphere [28,29,30]. The relationship between *Trichoderma*, host plants, and the environment is pivotal in understanding the mechanisms behind its beneficial effects on plant health [12,31]. However, the diversity of rhizosphere microbial communities associated with *Trichoderma*-treated plants is poorly understood. Gao et al. used high-throughput sequencing to analyze the effects of *T. viride* on the microbial community in soybean rhizosphere soil. The results showed that *T. viride* significantly reduced the soybean disease index to 15.11% and also affected the structure of the rhizosphere microbial community, increasing microbial diversity and reducing the relative abundance of pathogens [32]. Another study found that treating winter wheat with *Trichoderma atroviride* HB20111 significantly changed the composition and structure of the fungal community and improved yield, indicating its potential as a sustainable alternative for controlling wheat diseases [33]. Interestingly, a study showed that the application of the symbiotic fungus *Trichoderma asperellum* SL2 as a biofertilizer does not negatively impact soil bacterial populations; however, the authors also claimed that further research is needed to explore the effects of repeated applications over time on rice microbiota communities [34]. In this study, high-throughput sequencing of soybean rhizosphere soil treated with five *Trichoderma* strains indicated that certain treatments increased microbial diversity and species richness in the rhizosphere soil. As most *Trichoderma* species have the ability to combat pathogenic fungi, they can outcompete harmful pathogens for resources and space, effectively lowering *Fusarium* populations [35,36]. At the genus level, *Trichoderma* significantly reduced the relative abundance of plant pathogens such as *Cercospora*, *Fusarium*, and *Plectosphaerella* in the soil community, leading to various improvements in soybean yield. Additionally, reports indicate that the presence of diverse fungal communities can enhance plant resilience, as multiple microbial interactions may provide various forms of biological control and promote plant growth through improved nutrient uptake and stress alleviation [37,38]. Therefore, a comprehensive discussion on how *Trichoderma* influences fungal diversity in the rhizosphere and its correlation with the suppression of *Fusarium* is essential for developing effective biocontrol strategies and maximizing the benefits of these microorganisms in sustainable agriculture. Further research in this area could elucidate the specific interactions and mechanisms at play, offering new insights into leveraging *Trichoderma* for improved crop health and yields.

## 5. Conclusions

Our study highlights the effectiveness of *Trichoderma* spp. strains isolated from the soybean rhizosphere in promoting soybean growth and managing *Fusarium* root rot disease, successfully identifying multiple strains with antagonistic properties against *Fusarium* spp. that correlated with improved growth parameters and disease suppression in controlled greenhouse experiments. These findings indicate the potential for utilizing *Trichoderma* spp. as biocontrol agents in sustainable agricultural practices. However, this study has several limitations. The greenhouse conditions, while controlled, may not accurately replicate the complexities of field environments, where factors such as soil type, climate variability, and microbial interactions can influence the efficacy of *Trichoderma* spp. Additionally, the mechanisms underlying the beneficial effects of these strains on soybean health remain poorly understood, necessitating further investigation. The specific strains identified also need to be tested across a wider range of soybean varieties to assess their effectiveness comprehensively, and long-term studies are required to evaluate the stability of these biocontrol effects over multiple growing seasons.

## Figures and Tables

**Table 1:** The inhibitory effect of antagonistic strains on *Fusarium oxysporum* in soybeans on agar plates.

*Trichoderma* Strain	Growth Radius of *F. oxysporum* (cm)	IR
Control	2.63 ± 0.75 a	-
121A15	0.97 ± 0.31 bc	63.44 ± 0.02
134D16	0.83 ± 0.06 bc	66.88 ± 0.08
141F9	0.87 ± 0.06 bc	65.13 ± 0.11
215E14	1.10 ± 0.10 bc	56.29 ± 0.10
223H3	1.03 ± 0.25 bc	59.45 ± 0.11
223H16	1.00 ± 0.30 bc	62.15 ± 0.01
225J10	0.83 ± 0.15 bc	65.64 ± 0.16
231K4	0.83 ± 0.23 bc	67.96 ± 0.05
231K16	0.87 ± 0.25 bc	67.15 ± 0.02
234N4	1.33 ± 0.15 b	47.57 ± 0.10
235T4	0.67 ± 0.06 c	72.86 ± 0.10
243R6	0.90 ± 0.26 bc	65.70 ± 0.03
244S9	1.20 ± 0.53 bc	55.50 ± 0.08
311A2	0.93 ± 0.15 bc	63.64 ± 0.05
321F10	1.20 ± 0.26 bc	53.65 ± 0.06
324I4	1.17 ± 0.06 bc	53.55 ± 0.11
324I5	1.27 ± 0.38 b	51.69 ± 0.05
333C1	0.93 ± 0.23 bc	63.94 ± 0.06
333C17	1.17 ± 0.21 bc	54.16 ± 0.11
342G10	0.90 ± 0.30 bc	66.17 ± 0.02
343I8	0.87 ± 0.23 bc	66.85 ± 0.05
461C1	1.00 ± 0.17 bc	61.08 ± 0.06
415E2	0.87 ± 0.23 bc	63.75 ± 0.18
452B7	0.87 ± 0.38 bc	67.75 ± 0.06
551A6	0.87 ± 0.12 bc	65.90 ± 0.07
552B7	1.10 ± 0.26 bc	57.66 ± 0.05
553C1	0.90 ± 0.17 bc	63.68 ± 0.14
561A7	1.00 ± 0.26 bc	61.68 ± 0.04
563C3	1.07 ± 0.15 bc	58.34 ± 0.06
563C5	1.03 ± 0.25 bc	59.45 ± 0.11
611A17	0.97 ± 0.06 bc	60.81 ± 0.13
611A18	0.90 ± 0.20 bc	62.30 ± 0.19
612B8	0.93 ± 0.32 bc	64.72 ± 0.04
614D1	1.10 ± 0.20 bc	56.42 ± 0.13
623H1	0.80 ± 0.00 bc	67.87 ± 0.09
625J11	1.10 ± 0.46 bc	59.21 ± 0.05
651A8	0.80 ± 0.10 bc	68.64 ± 0.05

Note: Different letters indicate significant differences between different treatments (*p* < 0.05).

**Table 2:** Control effect of *Trichoderma* substrate on *Fusarium oxysporum* against soybean root rot disease.

Treatment	DI	RCE
Control	77.08	0
223H16	12.50	83.78
452B7	16.66	78.39
561A7	16.66	78.39
611A17	33.33	56.76
625J11	23.33	69.70

DI = Disease Indices; RCE = Relative Control Efficiencies.

**Table 3:** Effect of trifoliate stage *Trichoderma* substrate in field trials.

Treatment	Mengdou			Xingnong		
	PH (cm)	FW (g)	DW (g)	PH (cm)	FW (g)	DW (g)
Control	15.33 ± 0.68 b	2.38 ± 0.15 a	0.42 ± 0.00 b	14.57 ± 0.76 bc	2.10 ± 0.08 b	0.40 ± 0.01 b
223H16	16.23 ± 1.16 b	2.50 ± 0.32 a	0.45 ± 0.01 a	15.23 ± 0.47 b	2.37 ± 0.16 ab	0.43 ± 0.01 a
452B7	18.30 ± 0.56 a	2.43 ± 0.28 a	0.44 ± 0.01 a	13.73 ± 0.15 c	2.23 ± 0.23 ab	0.41 ± 0.01 ab
561A7	16.57 ± 0.61 b	2.36 ± 0.27 a	0.41 ± 0.00 b	13.90 ± 0.52 c	2.42 ± 0.25 ab	0.41 ± 0.01 ab
611A17	15.43 ± 0.31 b	2.37 ± 0.18 a	0.42 ± 0.00 b	16.63 ± 0.21 a	2.57 ± 0.11 a	0.42 ± 0.02 a
625J11	15.73 ± 0.45 b	2.15 ± 0.32 a	0.42 ± 0.01 b	17.0 ± 0.61 a	2.62 ± 0.41 a	0.43 ± 0.00 a

Note: The data in the table is the mean ± standard deviation; Different letters in the same column indicate significant differences between different treatments (*p* < 0.05). PH = Plant Height; FW = Fresh Weight; DW = Dry Weight.

**Table 4:** Effects of Trichoderma substrate at maturity in field experiments.

Treatment	Mengdou			Xingnong		
	PN	PH (cm)	HGW (g)	PN	PH (cm)	HGW (g)
Control	18.33 ± 0.58 d	56.67 ± 2.52 c	22.61 ± 0.21 cd	15.33 ± 1.53 c	62.67 ± 4.04 c	22.46 ± 0.21 bc
223H16	29.67 ± 4.04 a	72.33 ± 2.08 a	21.77 ± 0.43 d	26.33 ± 3.06 a	77.00 ± 2.00 a	21.52 ± 0.50 c
452B7	25.33 ± 4.93 abc	51.67 ± 2.52 d	23.70 ± 0.58 b	26.00 ± 3.00 a	64.67 ± 1.15 bc	24.35 ± 0.39 a
561A7	28.67 ± 2.52 ab	67.00 ± 1.73 b	24.81 ± 0.40 a	25.33 ± 2.08 ab	77.00 ± 2.65 a	21.83 ± 0.33 c
611A17	22.33 ± 1.15 cd	57.67 ± 1.15 c	23.85 ± 0.58 b	25.67 ± 2.08 ab	65.33 ± 0.58 bc	23.21 ± 1.02 b
625J11	24.00 ± 1.00 bc	55.00 ± 3.46 cd	23.22 ± 0.74 bc	21.67 ± 0.58 b	68.33 ± 2.31 b	24.92 ± 0.38 a

Note: The data in the table is the mean ± standard deviation; Different letters in the same column indicate significant differences between different treatments (*p* < 0.05). PH = Plant Height; FW = Fresh Weight; DW = Dry Weight.

**Table 5:** Fungal diversity indices under different treatments.

Treatment	Chao	Shannon
Control	512.01 ± 55.52 b	2.32 ± 0.12 b
223H16	638.25 ± 93.5 b	2.77 ± 0.32 b
452B7	674.61 ± 50.89 b	3.29 ± 0.43 a
561A7	625.58 ± 86.92 b	2.84 ± 0.34 b
611A17	705.9 ± 53.99 a	3.48 ± 0.54 a

Note: The data in the table is the mean ± standard deviation; Different letters in the same column indicate significant differences between different treatments (*p* < 0.05).

## Data Availability

The data that support the findings of this study are available from the corresponding author, Hongsheng Cai, Ph.D., upon reasonable request.

## Supplementary Materials

The following supporting information can be downloaded at https://www.mdpi.com/article/10.3390/jof11020159/s1, Figure S1: Antagonistic activity of five selective *Trichoderma* isolates (A. 223H16, B. 452B7, C. 561A7, D. 611A17, and E. 625J11) on mycelial growth using PDA; Figure S2: Colony morphological structures of antagonistic *Trichoderma*; Figure S3: Phylogenetic trees constructed based on ITS and TEF gene sequences; Table S1: Molecular sequences of *Trichoderma* strains used in this study; Table S2: Comparative analysis of species diversity and community coverage across different groups.
