# Supplementary material for: Screening of Antagonistic Trichoderma Strains to Enhance Soybean Growth
Source: J Fungi (Basel). 2025 Feb 19;11(2):159. doi: 10.3390/jof11020159 (PMC11856567; doi:10.3390/jof11020159)
Supplement: Supplementary file 1 [file jof-11-00159-s001.zip › List of supplementary figures and tables.pdf]

Supplementary Figure S1. Antagonistic activity of five selective *Trichoderma* isolates (A. 223H16, B. 452B7, C. 561A7, D. 611A17, and E. 625J11) on mycelial growth using PDA. From left to right, the isolates are tested against *F. oxysporum, F. solani, F. equiseti, F. proliferatum,* and *F. graminearum,* respectively.

Supplementary Figure S2. Colony morphological structures of antagonistic Trichoderma. A, B, C, D, E, F, G, H represent the following species: *T. atroviride, T. velutinum, T. brevicompactum, T. gamsii, T. hamatum, T. harzianum, T. afroharzianum, T. koningiopsis*. **1** illustrates the colony morphology of the antagonistic strains; **2** shows the mycelial morphology; **3** depicts the conidia.

Supplementary Figure S3. Phylogenetic trees constructed based on ITS and TEF gene sequences. **1** Phylogenetic tree constructed from ITS gene sequences; **2** Phylogenetic trees constructed from TEF gene sequences.

Supplementary Table S1. Molecular sequences of *Trichoderma* strains used in this study. This table presents the internal transcribed spacer (ITS) and translation elongation factor (TEF) sequences for *Trichoderma* strains.

Supplementary Table S2. Comparative analysis of species diversity and community coverage across different groups.
