# Supplementary material for: Screening of Antagonistic Trichoderma Strains to Enhance Soybean Growth
Source: J Fungi (Basel). 2025 Feb 19;11(2):159. doi: 10.3390/jof11020159 (PMC11856567; doi:10.3390/jof11020159)
Supplement: Supplementary file 1 [file jof-11-00159-s001.zip › Supplementary Figure S3.pdf]

A-1

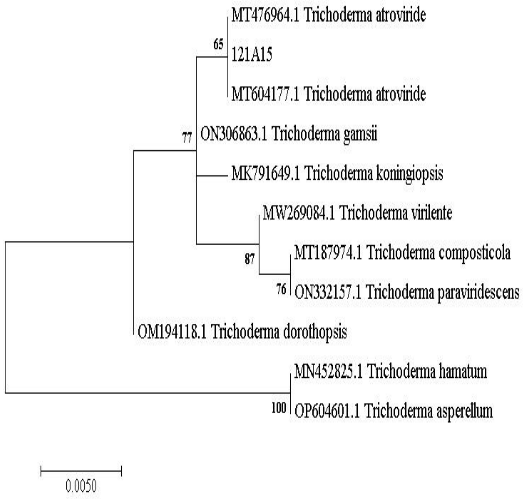
MT476964.1 Trichoderma atroviride
65
121A15
MT604177.1 Trichoderma atroviride
77
ON306863.1 Trichoderma gamsii
MK791649.1 Trichoderma koningiopsis
MW269084.1 Trichoderma virilente
87
MT187974.1 Trichoderma composticola
76
ON332157.1 Trichoderma paraviridescens
OM194118.1 Trichoderma dorothopsis
MN452825.1 Trichoderma hamatum
100
OP604601.1 Trichoderma asperellum
0.0050

A-2

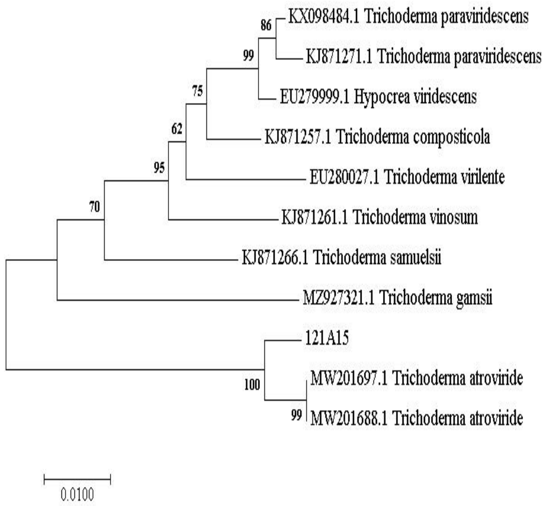
86
KX098484.1 Trichoderma paraviridescens
99
KJ871271.1 Trichoderma paraviridescens
75
EU279999.1 Hypocrea viridescens
62
KJ871257.1 Trichoderma composticola
95
EU280027.1 Trichoderma virilente
70
KJ871261.1 Trichoderma vinosum
KJ871266.1 Trichoderma samuelsii
MZ927321.1 Trichoderma gamsii
121A15
100
MW201697.1 Trichoderma atroviride
99
MW201688.1 Trichoderma atroviride
0.0100

B-1

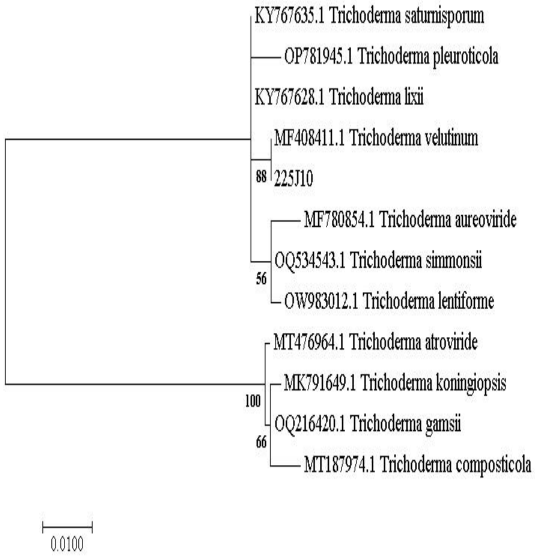
KY767635.1 Trichoderma saturnisporum
OP781945.1 Trichoderma pleuroticola
KY767628.1 Trichoderma lixii
MF408411.1 Trichoderma velutinum
88
225J10
MF780854.1 Trichoderma aureoviride
OQ534543.1 Trichoderma simmonsii
56
OW983012.1 Trichoderma lentiforme
MT476964.1 Trichoderma atroviride
MK791649.1 Trichoderma koningiopsis
100
OQ216420.1 Trichoderma gamsii
66
MT187974.1 Trichoderma composticola
0.0100

B-2

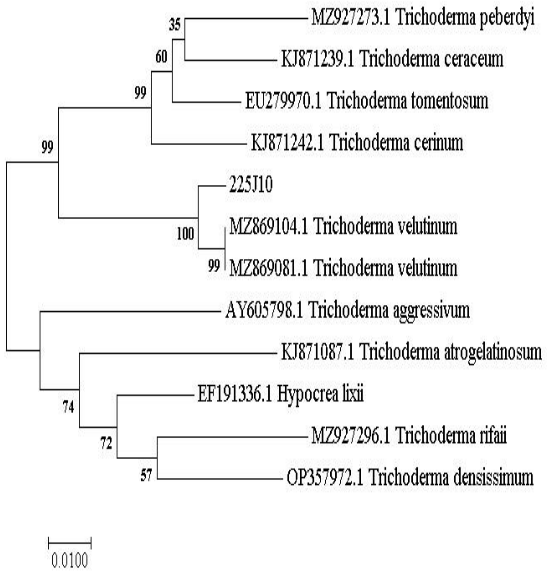
35
MZ927273.1 Trichoderma peberdyi
60
KJ871239.1 Trichoderma ceraceum
99
EU279970.1 Trichoderma tomentosum
99
KJ871242.1 Trichoderma cerinum
225J10
100
MZ869104.1 Trichoderma velutinum
99
MZ869081.1 Trichoderma velutinum
AY605798.1 Trichoderma aggressivum
KJ871087.1 Trichoderma atrogelatinosum
74
EF191336.1 Hypocrea lixii
72
MZ927296.1 Trichoderma rifaii
57
OP357972.1 Trichoderma densissimum
0.0100

C-1

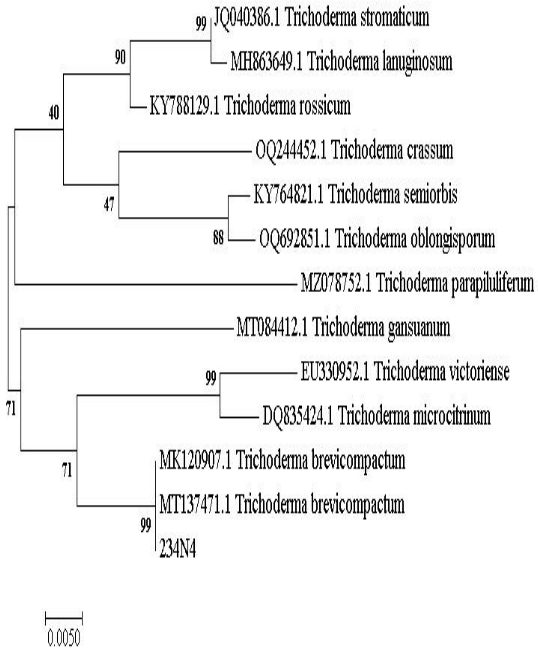
JQ040386.1 Trichoderma stromaticum
MH863649.1 Trichoderma lanuginosum
KY788129.1 Trichoderma rossicum
OQ244452.1 Trichoderma crassum
KY764821.1 Trichoderma semiorbis
OQ692851.1 Trichoderma oblongisporum
MZ078752.1 Trichoderma parapiluliferum
MT084412.1 Trichoderma gansuanum
EU330952.1 Trichoderma victoriense
DQ835424.1 Trichoderma microcitrinum
MK120907.1 Trichoderma brevicompactum
MT137471.1 Trichoderma brevicompactum
234N4
99
90
40
47
88
99
71
71
99
0.0050

C-2

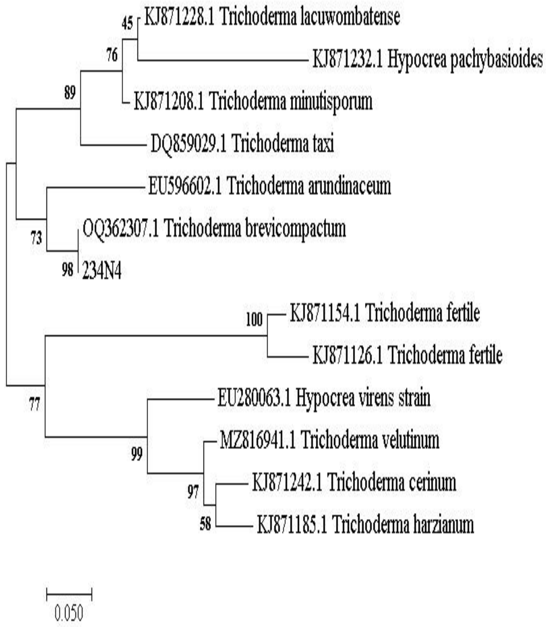
KJ871228.1 Trichoderma lacuwombatense
KJ871232.1 Hypocrea pachybasioides
KJ871208.1 Trichoderma minutisporum
DQ859029.1 Trichoderma taxi
EU596602.1 Trichoderma arundinaceum
OQ362307.1 Trichoderma brevicompactum
234N4
KJ871154.1 Trichoderma fertile
KJ871126.1 Trichoderma fertile
EU280063.1 Hypocrea virens strain
MZ816941.1 Trichoderma velutinum
KJ871242.1 Trichoderma cerinum
KJ871185.1 Trichoderma harzianum
45
76
89
73
98
100
77
99
97
58
0.050

D-1

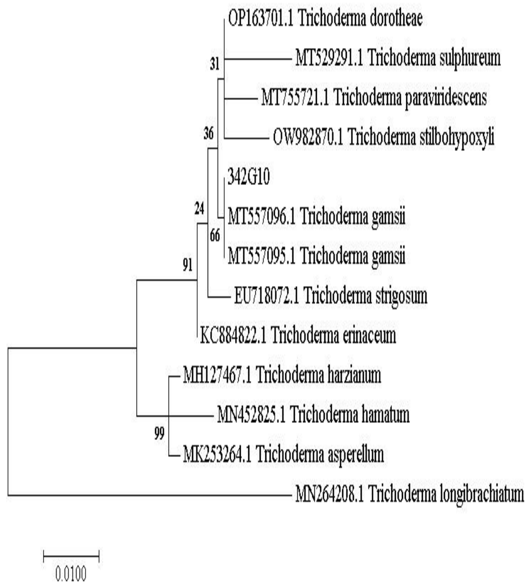
OP163701.1 Trichoderma dorotheae
MT529291.1 Trichoderma sulphureum
MT755721.1 Trichoderma paraviridescens
OW982870.1 Trichoderma stilbohypoxyli
342G10
MT557096.1 Trichoderma gamsii
MT557095.1 Trichoderma gamsii
EU718072.1 Trichoderma strigosum
KC884822.1 Trichoderma erinaceum
MH127467.1 Trichoderma harzianum
MN452825.1 Trichoderma hamatum
MK253264.1 Trichoderma asperellum
MN264208.1 Trichoderma longibrachiatum
31
36
24
66
91
99
0.0100

D-2

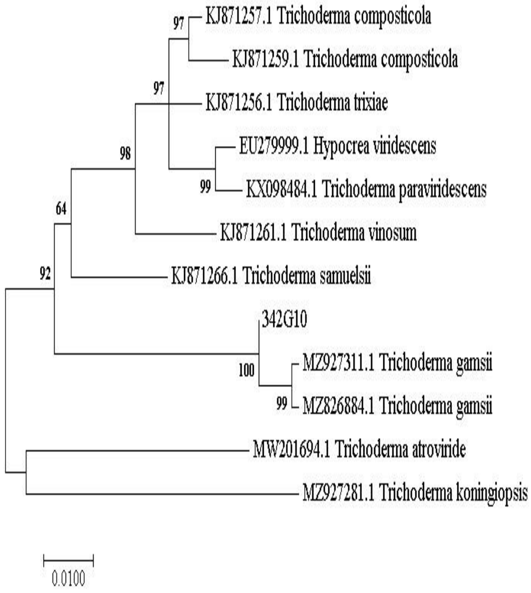
KJ871257.1 Trichoderma composticola
KJ871259.1 Trichoderma composticola
KJ871256.1 Trichoderma trixiae
EU279999.1 Hypocrea viridescens
KX098484.1 Trichoderma paraviridescens
KJ871261.1 Trichoderma vinosum
KJ871266.1 Trichoderma samuelsii
342G10
MZ927311.1 Trichoderma gamsii
MZ826884.1 Trichoderma gamsii
MW201694.1 Trichoderma atroviride
MZ927281.1 Trichoderma koningiopsis
97
97
98
99
64
92
100
99
0.0100

E-1

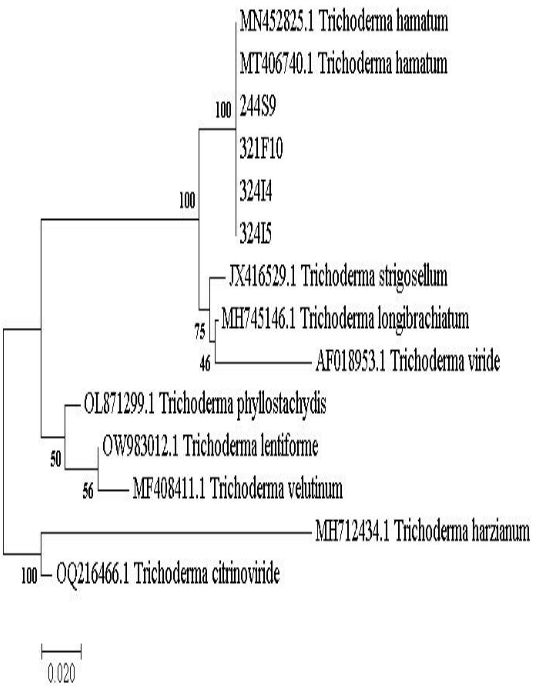
MN452825.1 Trichoderma hamatum
MT406740.1 Trichoderma hamatum
244S9
321F10
324I4
324I5
100
100
JX416529.1 Trichoderma strigosellum
MH745146.1 Trichoderma longibrachiatum
75
46
AF018953.1 Trichoderma viride
OL871299.1 Trichoderma phyllostachydis
OW983012.1 Trichoderma lentiforme
50
56
MF408411.1 Trichoderma velutinum
MH712434.1 Trichoderma harzianum
100
OQ216466.1 Trichoderma citrinoviride
0.020

E-2

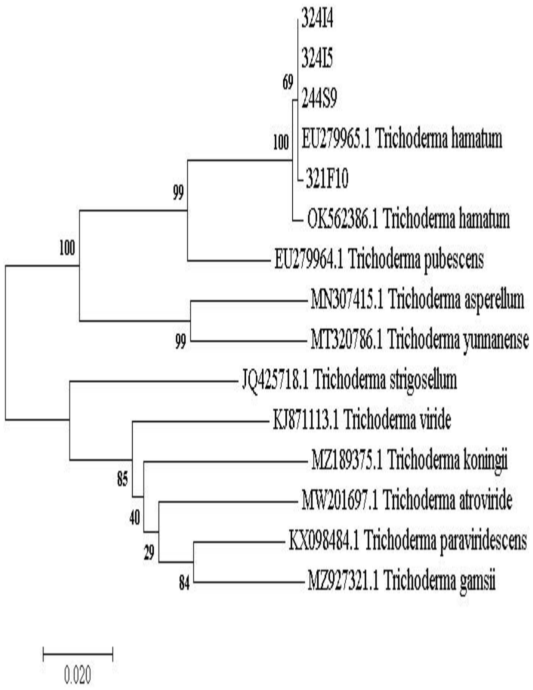
324I4
324I5
69
244S9
100
EU279965.1 Trichoderma hamatum
99
321F10
OK562386.1 Trichoderma hamatum
100
EU279964.1 Trichoderma pubescens
MN307415.1 Trichoderma asperellum
99
MT320786.1 Trichoderma yunnanense
JQ425718.1 Trichoderma strigosellum
KJ871113.1 Trichoderma viride
MZ189375.1 Trichoderma koningii
85
40
MW201697.1 Trichoderma atroviride
29
KX098484.1 Trichoderma paraviridescens
84
MZ927321.1 Trichoderma gamsii
0.020

F-1

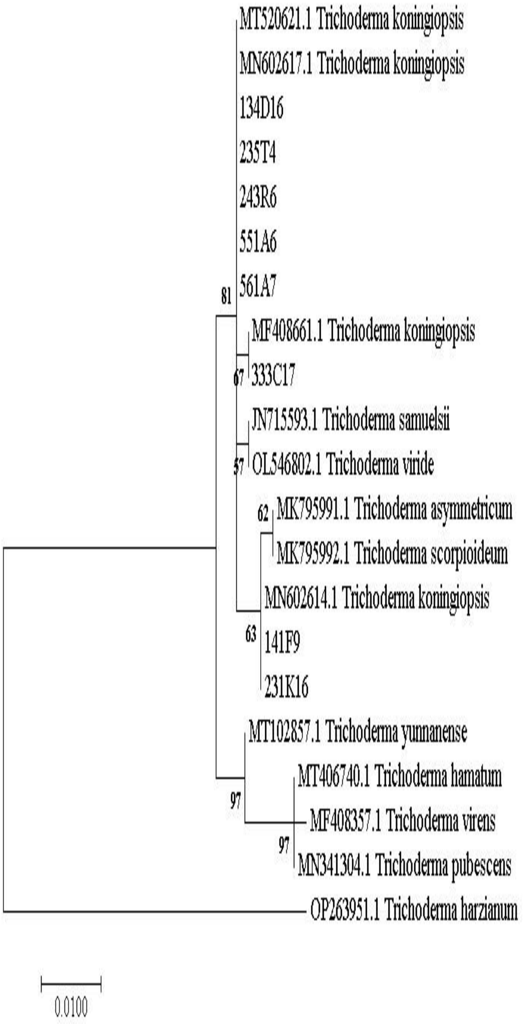
MT520621.1 Trichoderma koningiopsis
MN602617.1 Trichoderma koningiopsis
134D16
235T4
243R6
551A6
561A7
81
MF408661.1 Trichoderma koningiopsis
67
333C17
JN715593.1 Trichoderma samuelsii
57
OL546802.1 Trichoderma viride
62
MK795991.1 Trichoderma asymmetricum
MK795992.1 Trichoderma scorpioideum
MN602614.1 Trichoderma koningiopsis
63
141F9
231K16
MT102857.1 Trichoderma yunnanense
97
MT406740.1 Trichoderma hamatum
MF408357.1 Trichoderma virens
97
MN341304.1 Trichoderma pubescens
OP263951.1 Trichoderma harzianum
0.0100

F-2

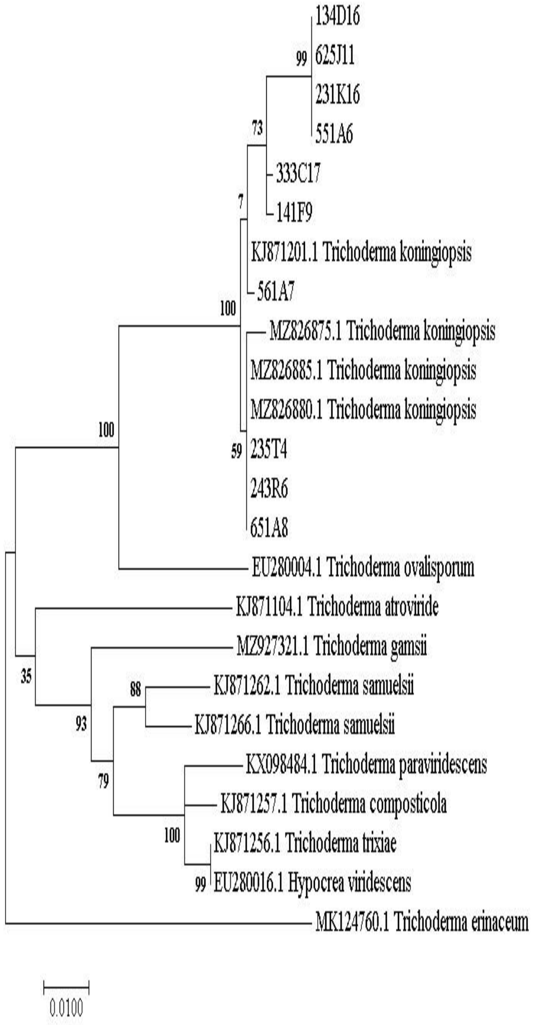
134D16
99
625J11
231K16
551A6
73
333C17
7
141F9
KJ871201.1 Trichoderma koningiopsis
561A7
100
MZ826875.1 Trichoderma koningiopsis
MZ826885.1 Trichoderma koningiopsis
MZ826880.1 Trichoderma koningiopsis
100
59
235T4
243R6
651A8
EU280004.1 Trichoderma ovalisporum
KJ871104.1 Trichoderma atroviride
MZ927321.1 Trichoderma gamsii
35
88
KJ871262.1 Trichoderma samuelsii
93
KJ871266.1 Trichoderma samuelsii
KX098484.1 Trichoderma paraviridescens
79
KJ871257.1 Trichoderma composticola
100
KJ871256.1 Trichoderma trixiae
99
EU280016.1 Hypocrea viridescens
MK124760.1 Trichoderma erinaceum
0.0100

G-1 G-2

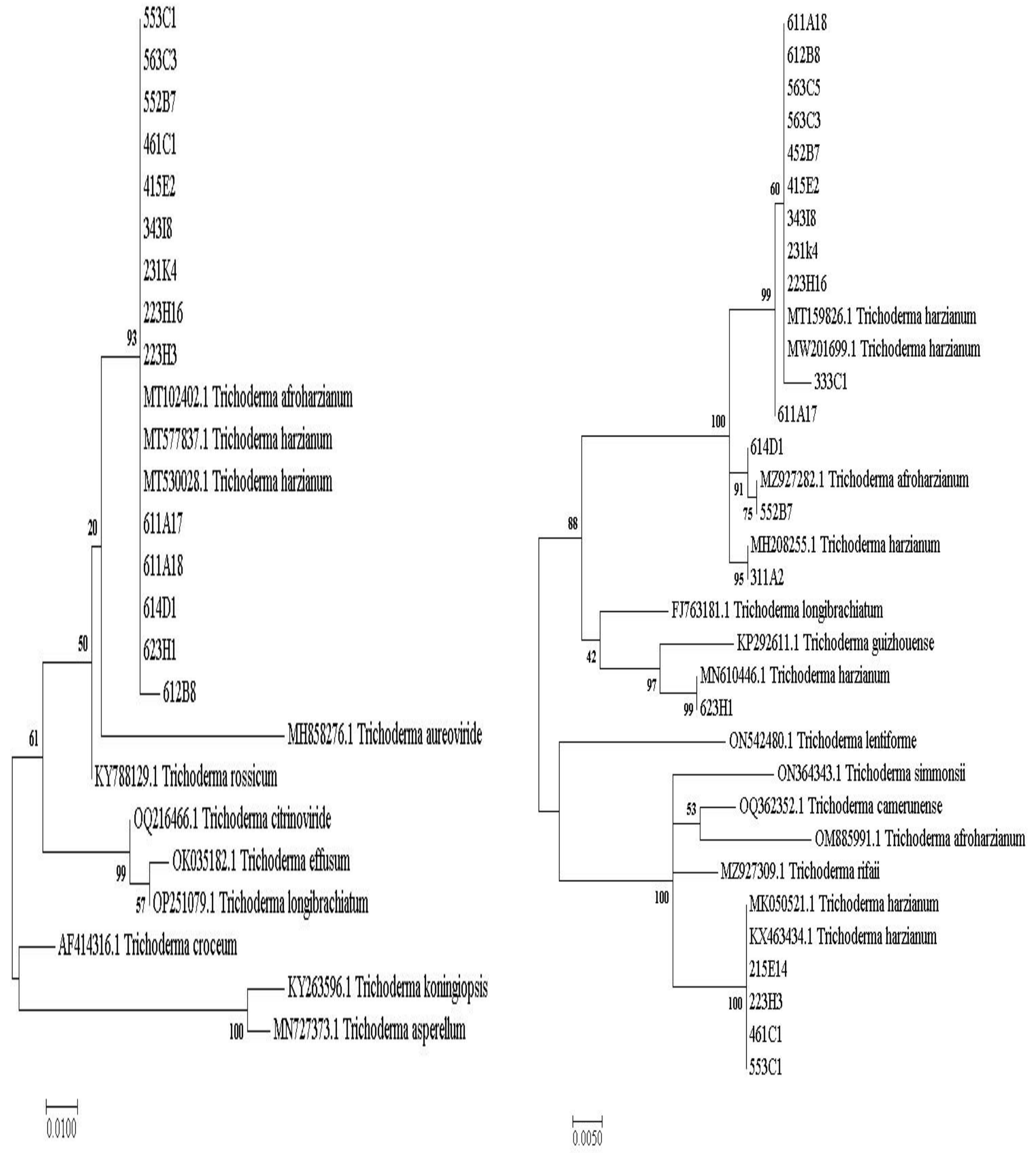

A Phylogenetic tree constructed based on ITS and TEF gene sequences

Note:-1:A Phylogenetic Tree Based on ITS Gene Sequence Construction; -2:A Phylogenetic Tree Based on TEF Gene Sequence Construction.
