# Supplementary material for: Screening of Antagonistic Trichoderma Strains to Enhance Soybean Growth
Source: J Fungi (Basel). 2025 Feb 19;11(2):159. doi: 10.3390/jof11020159 (PMC11856567; doi:10.3390/jof11020159)
Supplement: Supplementary file 1 [file jof-11-00159-s001.zip › Supplementary Table S1.pdf]

Supplementary Table S1. Molecular sequences of *Trichoderma* strains used in this study. This table presents the internal transcribed spacer (ITS) and translation elongation factor (TEF) sequences for Trichoderma strains.

Trichoderma strain	ITS sequence	TEF sequence
225J10	AACTGTTGCCTCGGCGGGATCTTCTGCCCCGGGTGCGTCGCAGCCCCGGACCAAGGCGCCC	CTGATTCTCAACACTTGTGCTAACTACCGTCTTCTAGGGGTGCGTATTTCATCAATCATCTTGAA
	GCCGGAGGAATCAACCAAAACTCTTATTGTATACCCCCTCGCGGGTTTTTTTATAATCTGAGCC	TGAGATCGATCGAACTCAATACTGACTTGCTACAACAGCCACGTCGACTCCGGCAAGTCGACC
	TTCTCGGCGCCTCTCGTAGGCGTTTCGAAAATGAATCAAAACTTTCAACAACGGATCTCTTGG	ACCGTGAGTTATACCCTCTTCTCTTACTCTGACATCACACGTTTTATCGTTTTGATGCGGAACAT
	TTCTGGCATCGATGAAGAACGCAGCGAAATGCGATAAGTAATGTGAATTGCAGAATTCAGTGA	CTACTCTTCGAACATAGAGCTAACCATCTATCATACAGACTGGTCACTTGATCTACCAGTGCGG
	ATCATCGAATCTTTGAACGCACATTGCGCCCGCCAGTATTCTGGCGGGCATGCCTGTCCGAGC	TGGTATCGACAAGCGTACCATCGAGAAGTTCGAGAAGGTAAGCTTCATCAACTGATTTTCGCC
	GTCATTTCAACCCTCGAACCCCTCCGGGGGGTCGGCGTTGGGGATCGGCCCTCCTCTTGCGG	TCGATTTCCCCTTCACATTCAATTGTGCCCGACAATTCTGAAAAGAACTTTCGTGTCAACAATT
	GGGCCGTCTCCGAAATACAGTGGCGGTCTCGCCGCAGCCTCTCCTGCGCAGTAGTTTGCACA	TTTCGTCACCCCGCTTTCCATTACCCCTCCTTTGCAGCGACGCAAATTTTTTTGCTGTCTTTTG
	CTCGCATCGGGAGCGCGGCGCGTCCACAGCCGTTAAACACCCAACTTCTGAAATGTTGACCT	GTTTTAGTGGGGTTCCCTGCGCACCCCACTAGCTAACTGCTTTTCTGTGCTTCACTCTCACTGC
	CGGATCAGGTAGGAATACCCGCTGAACTTAAGCATATCAATAAGCGGAG	CTAGCCAACATTAAACGTGCTCTGTATCCCGTTACTTTCAACGATGCTAACCACTTTCTCATCAA
		TAGGAAGCCGCTGAACTCGGCAAGGGTTCCTTCAAGTACGCATGGGTTCTTGACAAGCTCAA
		GGCCGAGCGTGAGCGTGGTATCACCATCGACATTGCCCTCTGGAAGTTCGAGACTCCCAAGT
		ACTATGTCACCGTCATTGGTATGTCTGATTCATCAACTTGATGCAGCAATTGCAAGTCAGTGCT
		AACAGATGTTTCATAGACGCCCCCG
234N4	CCTCGGCGGGATTTCTGCCCCGGGCGCGTCGCAGCCCCGGACCAAGGCGCCCGCCGGAGG	AATATTCAAGAGTGTCTCGTCTTCTGCTCCTTACCCGTTTTGCTCTTGTGCTAACCATCATCGTC
	ACCAATTTACAAACTCTTTTGTATATCCCATCGCGGATTCTTTACATTCTGAGCTTTCTCGGCGC	TTCTAGGGGTGCGTATTTCCATCAATCCATCTCCGTTCAGATTGAACTCGATCGAACTCAATACT
	TCCTAGCGAGCGTTTCGAAAATGAATCAAAACTTTCAACAACGGATCTCTTGGTTCTGGCATC	GACTTGCTACAACAGCCACGTCGACTCCGGCAAGTCGACCACCGTGAGTTATACCCGTTTCTC
	GATGAAGAACGCAGCGAAATGCGATAAGTAATGTGAATTGCAGAATTCAGTGAATCATCGAAT	TGCCCTCCGACAACACTTCTGTTGCCGCGAGGTATCTCTTTTGAACACACCAAGCTAATATTTG
	CTTTGAACGCACATTGCGCCCGCCAGTATTCTGGCGGGCATGCCTGTCCGAGCGTCATTTCAA	CTCTATAGACTGGTCACTTGATCTACCAGTGCGGTGGTATCGACAAGCGTACCATCGAGAAGT
	CCCTCGAACCCCTCCGGGGGGTCGGCGTTGGGGATCGGCACTTACCTGCCGGCCCCGAAATA	TCGAGAAGGTAAGCTTACGCCTGATTTTCTCTCTCATTTTGACCTTCACTCCATGCAATTGTGC
	CAGTGGCGGTCTCGCCGCAGCCTCTCCTGCGCAGTAGTTTGCACACTCGCACCGGGAGCGCG	CCGACAATTCTCAGTTATCTGAGGATTTCGTGTCAATTTTTTTTCTTCATGGTCACCCCGCTTTC
	GCGCGTCCACGGCCGTAAAACAACCCAAACTTCTGAAATGTGACCTCGGATCAGGTAG	GCGGCCTACCCCTCCTTTGGCACAGACCTGCAAAATTTTTTTTGCTGCCTTACTAGGTTTTAGT
		GGGGGTACTTCTTGGAGCAAAACCCCACTACTACCGGTGACGCTCTGTTACCTGTT
342G10	CAAACTGTTGCCTCGGCGGGGTCACGCCCCGGGTGCGTCGCAGCCCCGGAACCAGGCGCCC	CACTGATGTGTATGACATTTTGCTAACAATCATCGTCTAGGGGTTCGTATTTCTCCATCAGACA
	GCCGGAGGGACCAACCAAACTCTTTTCTGTAGTCCCCTCGCGGACGTTATTTCTTACAGCTCT	GCTTTCGATCGACTCAAATACTGACTTGCTACAACAGCCACGTCGACTCCGGCAAGTCTACCA
	GAGCAAAAATTCAAAATGAATCAAAACTTTCAACAACGGATCTCTTGGTTCTGGCATCGATGA	CCGTGAGTAACTCCCAATTCCTCGAGCCCAGTTGCCATCGACTCTGTCGGTCGGCGCGGGGT
	AGAACGCAGCGAAATGCGATAAGTAATGTGAATTGCAGAATTCAGTGAATCATCGAATCTTTG	ATCTCATCATGAATGCATCCGGCTGACATTCCAATAGACTGGTCACTTGATCTACCAGTGCGGT
	AACGCACATTGCGCCCGCCAGTATTCTGGCGGGCATGCCTGTCCGAGCGTCATTTCAACCCTC	GGTATTGACAAGCGTACCATCGAGAAGTTCGAGAAGGTAAGCTAATTTCACTATTTTTATCACT
	GAACCCCTCCGGGGGGTCGGCGTTGGGGATCGGGAACCCCTAAGACGGGATCCCGGCCCCG	ACGCTTTATTGGCACAGTCGTGCGTCCGACAATCCTGTTCTCAGTCTTGTCAATTTTTCTCTCG
	AAATACAGTGGCGGTCTCGCCGCAGCCTCTCCTGCGCAGTAGTTTGCACAACTCGCACCGGG	CATCGTCACACCCCGCTCTACCTGTCTCTACCCCTCCTTTGGCACAGCAAAAATTTTCTGGCTG
	AGCGCGGCGCGTCCACGTCCGTAAAACACCCAACTTCTGAAATGTTGACCTCGGATCAGGTA	CCTTGTTTGGTTTTTAGTGGGGTGCCAGCTTTTTTTTTCTGGCAACCCCGCTAATCGCCGCTG
	GGAATACCCGCTGAAC	TCCCTCATCCATCGTCTTAACAATTTGTTCACTCAATCGCATCTCATTTTCTCCGTGGTTCAATGT
		GCTGATCATGATTCAATCAATAGGAAGCCGCCGAACTCGGCAAGGGTTCTTTCAAGTATGCGT
		GGGTTCTTGACAAGCTCAAGGCCGAGCGTGAGCGTGGTATCACCATCGACATTGCCCTCTGG
		AAGTTCGAGACTCCCAAGTACTATGTCACCGTCATTGGTATGTTTTAGTATCCTCATTGGCGTTT
		CGAAATCATGATTCTAACGTGCCACTCTACAGACGCTCCCGGCCACCGTGATTTCAT
121A15	ACCATACCAAACTGTTGCCTCGGCGGGGTCACGCCCCGGGTGCGTCGCAGCCCCGGAACCA	CTGCCGTTGACACTGCTGTGTATGACATTTTGCTGACCATCATCGTCTAGGGGTTCGTATTTCT
	GGCGCCCGCCGGAGGGACCAACCAAACTCTTTTCTGTAGTCCCCTCGCGGACGTTATTTCTTA	CCATCAGACAGCTTTCGATCGACTCCAATACTGACTTGCTACAACAGCCACGTCGACTCCGGC
	CAGCTCTGAGCAAAAATTCAAAATGAATCAAAACTTTCAACAACGGATCTCTTGGTTCTGGCA	AAGTCTACCACCGTGAGTAACTCCCAATCCCTCGAGCCCTACAGCCATCGACTCTGTCGGTCG
	TCGATGAAGAACGCAGCGAAATGCGATAAGTAATGTGAATTGCAGAATTCAGTGAATCATCGA	GCGCGGGGTATTATCATCATCATGAACGCATCCAGCTGACATTTTCCCAACAGACTGGTCACTT
	ATCTTTGAACGCACATTGCGCCCGCCAGTATTCTGGCGGGCATGCCTGTCCGAGCGTCATTTC	GATCTACCAGTGCGGTGGTATTGACAAGCGTACCATCGAGAAGTTCGAGAAGGTGAGCTCAT

	AACCCTCGAACCCCTCCGGGGGGTCGGCGTTGGGGACCTCGGGAGCCCCTAAGACGGGATC<br>CCGGCCCCGAAATACAGTGGCGGTCTCGCCGCAGCCTCTCCTGCGCAGTAGTTTGCACAACT<br>CGCACCGGGAGCGCGGCGCGTCCACGTCCGTAAAACACCCAACTTTCTGAAATGTTGACCTC<br>GGATCAGGTAGGAATACCCGCTGAACTTAAGC	TTCTGCTTTTTCACTCCGCTCCCTGAGCACAATCGTGCCCGACAATTCTGTCCTCAGTCTTGTC<br>ATTTTTTTTCCTCGCAGCATCACACCCCGCTTTACCTGTCTACCCCTCCTTTGGCACAGCAAAA<br>TTTTCTGGCGGCCTTGCTTGGCTTTTAGTGGGGTGCCAACTTTTTTTTGTTTGGCTGCAACCC<br>CGCTATCGCCACTGTCCCGTCCCAACGAATTGTACTCAATTGCATCGTCTTCTGCTGGTTCATT<br>GTGCTAATCATGCTTCAATCAATAGGAAGCCGCCGAACTCGGCAAGGGTTCTTTCAAGTATGC<br>GTGGGTTCTTGACAAGCTCAAGGCCGAGCGTGAGCGTGGTATCACCATCGACATTGCCCTCT<br>GGAAGTTCGAGACTCCCAAGTACTATGTCACCGTCATTGGTATGTTTCCGCTTTTCCTCATTGA<br>CCCTTTGAGACCATCATTCTAACGTGTCGCTCTGCAGACGCTCCCGG
244S9	CGGCGGGGTCACGCCCCGGGTGCGTAAAAGCCCCGGAACCAGGCGCCCGCCGGAGGAACC<br>AACCAAACTCTTTCTGTAGTCCCCTCGCGGACGTATTTCTTACAGCTCTGAGCAAAAATTCAAA<br>ATGAATCAAAACTTTCAACAACGGATCTCTTGGTTCTGGCATCGATGAAGAACGCAGCGAAAT<br>GCGATAAGTAATGTGAATTGCAGAATTCAGTGAATCATCGAATCTTTGAACGCACATTGCGCC<br>CGCCAGTATTCTGGCGGGCATGCCTGTCCGAGCGTCATTTCAACCCTCGAACCCCTCCGGGG<br>GATCGGCGTTGGGGATCGGGACCCCTCACCGGGTGCCGGCCCTGAAATACAGTGGCGGTCT<br>CGCCGCAGCCTCTCCTGCGCAGTAGTTTGCACAACTCGCACCGGGAGCGCGGCGCGTCCAC<br>GTCCGTAAAACACCCAACTTCTGAAATGTTGACCTCGGATCAGGTAGGAATACCCGCTGAACT<br>TA	CCCATTCTTCTCTTCAGCGGCATTCTCTGCTGCTGGCTGTGAATGACATCGTGCTAACCGTCAT<br>CTTCTAGGGGTTCGTATTTCTCCATCAAACTGCTTTCGATCGACTCCAATACTGACTTGCTACA<br>ACAGCCACGTCGACTCCGGCAAGTCTACCACCGTGAGTAATATCCAATTCCTCAAGCCTCTCT<br>GCCATCGGCTCTGTCGGTCGGCGCGGGGTATATCAAGTATGAACGCATCAAGCTAACGTTTTC<br>CAATAGACTGGTCACTTGATCTACCAGTGCGGTGGTATTGACAAGCGTACCATCGAGAAGTTC<br>GAGAAGGTAAGTTCATTTCGCTGCTTTTTTTATTCCTTTTGGGCACAATTGTGCCAGACAATTC<br>TGTTCTCAGTCTTGTCAACATTTTTTCCCACCAAGCATCGCACCCCGCTTTGTCTGCCTACCTAC<br>CCCTCCTTTGGCACAGCAAAAATTTTCTGGCTGCCTTGGTTGGTTTTTAGTGGGGTGCCAAAT<br>TTTTGGCAGTGACCCCGCCATCGCCACTGTTCCTCATGCACTACCCAACACATGCTACGTATCA<br>ACTGCTTGGTTCATTGTGCTAATCATACTTCAATCAATAGGAAGCCGCCGAACTCGGCAAGGG<br>TTCCTTCAAGTATGCGTGGGTTCTTGACAAGCTCAAGGCCGAGCGTGAGCGTGGTATCACCA<br>TCGACATTGCCCTGTGGAAGTTCGAGACTCCCAAGTACTATGTCACCGTCATTGGTATGTTTTC<br>AGTCCGACTGGTCACTATCCCAACATCATCATGCTAACGTGCGACTCCACAGACGCTCCC
321F10	ACTGTTGCCTCGGCGGGGTCACGCCCCGGGTGCGTAAAAGCCCCGGAACCAGGCGCCCGCC<br>GGAGGAACCAACCAAACTCTTTCTGTAGTCCCCTCGCGGACGTATTTCTTACAGCTCTGAGCA<br>AAAATTCAAAATGAATCAAAACTTTCAACAACGGATCTCTTGGTTCTGGCATCGATGAAGAAC<br>GCAGCGAAATGCGATAAGTAATGTGAATTGCAGAATTCAGTGAATCATCGAATCTTTGAACGC<br>ACATTGCGCCCGCCAGTATTCTGGCGGGCATGCCTGTCCGAGCGTCATTTCAACCCTCGAACC<br>CCTCCGGGGGATCGGCGTTGGGGATCGGGACCCCTCACCGGGTGCCGGCCCTGAAATACAG<br>TGGCGGTCTCGCCGCAGCCTCTCCTGCGCAGTAGTTTGCACAACTCGCACCGGGAGCGCGGC<br>GCGTCCACGTCCGTAAAACACCCAACTTCTGAAATGTTGACCTCGGATCAGGTAGGAATACCC<br>GCTGAAC	GCATTCTCTGCTGCTGGTTGTGAATGACATCGTGCTAACCGTCATCTTCTAGGGGTTCGTATTT<br>CTCCATCAAACTGCTTTCGATCGACTCCAATACTGACTTGCTACAACAGCCACGTCGACTCCG<br>GCAAGTCTACCACCGTGAGTAATATCCAATTCCTCAAGCCTCTCTGCCATCGGCTCTGTCGGTC<br>GGCGCGGGGTATATCAAGTATGAACGCATCAAGCTAACGTTTTCCAATAGACTGGTCACTTGA<br>TCTACCAGTGCGGTGGTATTGACAAGCGTACCATCGAGAAGTTCGAGAAGGTAAGTTCATTTC<br>GCTGCTTTTTTTATTCCTTTTGGGCACAATTGTGCCAGACAATTCTGTTCTCAGTCTTGTCAAC<br>ATTTTTTCCCACCAAGCATCGCACCCCGCTTTGTCTGCCTACCTACCCCTCCTTTGGCACAGCA<br>AAAATTTTCTGGCTGCCTTGGTTGGTTTTTAGTGGGGTGCCAAATTTTTGGCAGTGACCCCGC<br>CATCGCCACTGTTCCTCATGCACTACCCAACACATGCTACGTATCAACTGCTTGGTTCATTGTG<br>CTAATCATACTTCAATCAATAGGAAGCCGCCGAACTCGGCAAGGGTTCCTTCAAGTATGCGTG<br>GGTTCTTGACAAGCTCAAGGCCGAGCGTGAGCGTGGTATCACCATCGACATTGCCCTGTGGA<br>AGTTCGAGACTCCCAAGTACTATGTCACCGTCATTGGTATGTTTTCAGTCCGACTGGTCACTAT<br>CCCAACATCATCATGCTAACGTGCGACTCCACAGACGCTCCCGGT
324I4	ACTGTTGCCTCGGCGGGGTCACGCCCCGGGTGCGTAAAAGCCCCGGAACCAGGCGCCCGCC<br>GGAGGAACCAACCAAACTCTTTCTGTAGTCCCCTCGCGGACGTATTTCTTACAGCTCTGAGCA<br>AAAATTCAAAATGAATCAAAACTTTCAACAACGGATCTCTTGGTTCTGGCATCGATGAAGAAC<br>GCAGCGAAATGCGATAAGTAATGTGAATTGCAGAATTCAGTGAATCATCGAATCTTTGAACGC<br>ACATTGCGCCCGCCAGTATTCTGGCGGGCATGCCTGTCCGAGCGTCATTTCAACCCTCGAACC<br>CCTCCGGGGGATCGGCGTTGGGGATCGGGACCCCTCACCGGGTGCCGGCCCTGAAATACAG<br>TGGCGGTCTCGCCGCAGCCTCTCCTGCGCAGTAGTTTGCACAACTCGCACCGGGAGCGCGGC<br>GCGTCCACGTCCGTAAAACACCCAACTTCTGAAATGTTGACCTCGGATCAGGTAGGAATACCC<br>GCTGAACTTA	GTTGCAATCCCATTCTTCTCTTCAGCGGCATTCTCTGCTGCTGGCTGTGAATGACATCGTGCTA<br>ACCGTCATCTTCTAGGGGTTCGTATTTCTCCATCAAACTGCTTTCGATCGACTCCAATACTGACT<br>TGCTACAACAGCCACGTCGACTCCGGCAAGTCTACCACCGTGAGTAATATCCAATTCCTCAAG<br>CCTCTCTGCCATCGGCTCTGTCGGTCGGCGCGGGGTATATCAAGTATGAACGCATCAAGCTAA<br>CGTTTTCCAATAGACTGGTCACTTGATCTACCAGTGCGGTGGTATTGACAAGCGTACCATCGA<br>GAAGTTCGAGAAGGTAAGTTCATTTCGCTGCTTTTTTTATTCCTTTTGGGCACAATTGTGCCA<br>GACAATTCTGTTCTCAGTCTTGTCAACATTTTTTCCCACCAAGCATCGCACCCCGCTTTGTCTG<br>CCTACCTACCCCTCCTTTGGCACAGCAAAAATTTTCTGGCTGCCTTGGTTGGTTTTTAGTGGG<br>GTGCCAAATTTTTGGCAGTGACCCCGCCATCGCCACTGTTCCTCATGCACTACCCAACACATG<br>CTACGTATCAACTGCTTGGTTCATTGTGCTAATCATACTTCAATCAATAGGAAGCCGCCGAACT

		CGGCAAGGGTTCCTTCAAGTATGCGTGGGTTCTTGACAAGCTCAAGGCCGAGCGTGAGCGTGGTATCACCATCGACATTGCCCTGTGGAAGTTCGAGACTCCCAAGTACTATGTCACCGTCATTGGTATGTTTTCAGTCCGACTGGTCACTATCCCAACATCATCATGCTAACGTGCGACTCCACAGACGCTCCCGGTCACCGTGATT
324I5	CTCGGCGGGGTCACGCCCCGGGTGCGTAAAAGCCCCGGAACCAGGCGCCCGCCGGAGGAACCAACCAAACTCTTTCTGTAGTCCCCTCGCGGACGTATTTCTTACAGCTCTGAGCAAAAATTCAAAATGAATCAAAACTTTCAACAACGGATCTCTTGGTTCTGGCATCGATGAAGAACGCAGCGAAATGCGATAAGTAATGTGAATTGCAGAATTCAGTGAATCATCGAATCTTTGAACGCACATTGCGCCCGCCAGTATTCTGGCGGGCATGCCTGTCCGAGCGTCATTTCAACCCTCGAACCCCTCCGGGGGATCGGCGTTGGGGATCGGGACCCCTCACCGGGTGCCGGCCCTGAAATACAGTGGCGGTCTCGCCGCAGCCTCTCCTGCGCAGTAGTTTGCACAACTCGCACCGGGAGCGCGGCGCGTCCACGTCCGTAAAACACCCAACTTCTGAAATGTTGACCTCGGATCAGGTAGGAATACCCGCTG	TCCCATTCTTCTCTTCAGCGGCATTCTCTGCTGCTGGCTGTGAATGACATCGTGCTAACCGTCATCTTCTAGGGGTTCGTATTTCTCCATCAAACTGCTTTCGATCGACTCCAATACTGACTTGCTACAACAGCCACGTCGACTCCGGCAAGTCTACCACCGTGAGTAATATCCAATTCCTCAAGCCTCTCTGCCATCGGCTCTGTCGGTCGGCGCGGGGTATATCAAGTATGAACGCATCAAGCTAACGTTTTCCAATAGACTGGTCACTTGATCTACCAGTGCGGTGGTATTGACAAGCGTACCATCGAGAAGTTCGAGAAGGTAAGTTCATTTCGCTGCTTTTTTTATTCCTTTTGGGCACAATTGTGCCAGACAATTCTGTTCTCAGTCTTGTCAACATTTTTTCCCACCAAGCATCGCACCCCGCTTTGTCTGCCTACCTACCCCTCCTTTGGCACAGCAAAAATTTTCTGGCTGCCTTGGTTGGTTTTTAGTGGGGTGCCAAATTTTTGGCAGTGACCCCGCCATCGCCACTGTTCCTCATGCACTACCCAACACATGCTACGTATCAACTGCTTGGTTCATTGTGCTAATCATACTTCAATCAATAGGAAGCCGCCGAACTCGGCAAGGGTTCCTTCAAGTATGCGTGGGTTCTTGACAAGCTCAAGGCCGAGCGTGAGCGTGGTATCACCATCGACATTGCCCTGTGGAAGTTCGAGACTCCCAAGTACTATGTCACCGTCATTGGTATGTTTTCAGTCCGACTGGTCACTATCCCAACATCATCATGCTAACGTGCGACTCCACAGACGCTCCCGGTCACCGTGATTTCATCAAG
551A6	CCATACCAAACTGTTGCCTCGGCGGGGTCACGCCCCGGGTGCGTCGCAGCCCCGGAACCAGGCGCCCGCCGGAGGGACCAACCAAACTCTTTCTGTAGTCCCCTCGCGGACGTTATTTCTTACAGCTCTGAGCAAAAATTCAAAATGAATCAAAACTTTCAACAACGGATCTCTTGGTTCTGGCATCGATGAAGAACGCAGCGAAATGCGATAAGTAATGTGAATTGCAGAATTCAGTGAATCATCGAATCTTTGAACGCACATTGCGCCCGCCAGTATTCTGGCGGGCATGCCTGTCCGAGCGTCATTTCAACCCTCGAACCCCTCCGGGGGGTCGGCGTTGGGGATCGGGAACCCCTAAGACGGGATCCCGGCCCCGAAATACAGTGGCGGTCTCGCCGCAGCCTCTCCTGCGCAGTAGTTTGCACAACTCGCACCGGGAGCGCGGCGCGTCCACGTCCGTAAAACACCCAACTTCTGAAATGTTGACCTCGGATCAGGTAGGAATACCCGCTGAACTTAAGCATATCAATAAGCGGAGGAA	CGGCACTCTCTATCTGCCGTTGACGCTGATGCGTATGACATCTTGCTGACCATAATCGTCTAGGGGTTCGTATTTTTCCATCAGACAGCTTTCGACCGACTTCAATACTGACTTGCTACAACAGCCACGTCGACTCCGGCAAGTCTACCACCGTGAGTAACTCCCAATTCCTCGAGCCCTGCTGCCATTGACTCTGTCGGTCGGCGCGGGGTATCTTGACCTTGAACGCATCCAGCTAACATTTTCCCAATAGACTGGTCACTTGATCTACCAGTGCGGTGGTATTGACAAGCGTACCATCGAGAAGTTCGAGAAGGTAAGCTCATTTCACTACTTTTTCTACCACGCTTGGCACAATTGTGCCCGACAATTCTGTTCTCAGTCTTGTCTGTTTACCCTCGCAGCGTCACACCCCGCTTGGCCTGTCTACCCCTCCTTTGGCAGCAAATTTTTCTGCTGCCTCGTTTGACTTTAGTGGGGTGTCAATTTTTTTGGCAACCCCCGCTATCGCCACTGTCCCTCATCAATCGTCCCAACAAAATGCACTCATTCAATCGCATCGTCTTTTGACTCGATTTCTCTGTGGTTCGTTGTGCTAATCATGCTTCAATCAATAGGAAGCCGCCGAACTCGGCAAGGGTTCCTTCAAGTATGCGTGGGTTCTTGACAAGCTCAAGGCCGAGCGTGAGCGTGGTATCACCATCGACATTGCCCTCTGGAAGTTCGAGACTCCCAAGTACTATGTCACCGTCATTGGTATGTTATTCCTGGCTCTTGACATGTCGAAATCATCATTCTAACGTGCCAATACAGACGCTCCCGGCCACCGTGATT
235T4	CTGTTGCCTCGGCGGGGTCACGCCCCGGGTGCGTCGCAGCCCCGGAACCAGGCGCCCGCCGGAGGGACCAACCAAACTCTTTCTGTAGTCCCCTCGCGGACGTTATTTCTTACAGCTCTGAGCAAAAATTCAAAATGAATCAAAACTTTCAACAACGGATCTCTTGGTTCTGGCATCGATGAAGAACGCAGCGAAATGCGATAAGTAATGTGAATTGCAGAATTCAGTGAATCATCGAATCTTTGAACGCACATTGCGCCCGCCAGTATTCTGGCGGGCATGCCTGTCCGAGCGTCATTTCAACCCTCGAACCCCTCCGGGGGGTCGGCGTTGGGGATCGGGAACCCCTAAGACGGGATCCCGGCCCCGAAATACAGTGGCGGTCTCGCCGCAGCCTCTCCTGCGCAGTAGTTTGCACAACTCGCACCGGGAGCGCGGCGCGTCCACGTCCGTAAAACACCCAACTTCTGAAATGTTGACCTCGGATCAGGTAGGAATACCCGCTGAACTTAAGCATATCAATAAGCCGGAGGAA	ATTCTCGTCATCGGCACTCTCTATCTGCCGTTGACGCTGATGCGTATGACATCTTGCTGACCATAATCGTCTAGGGGTTCGTATTTTTCCATCAGACAGCTTTCGACCGACTTCAATACTGACTTGCTACAACAGCCACGTCGACTCCGGCAAGTCTACCACCGTGAGTAACTCTCAATTCCTCGAGCCCTGCTGCCATTGACTCTGTCGGTCGGCGCGGGGTATCTTGATCTTGAACGCATCCAGCTAACATTTTCCCAATAGACTGGTCACTTGATCTACCAGTGCGGTGGTATTGACAAGCGTACCATCGAGAAGTTCGAGAAGGTAAGCTCATTTCACTACTTTTCCCACCACGCTTGGCACAATCGTGTCCGACAATTCTGTTCTCAGTCTTGTCTGTTTTCCTCGCAGCGTCACACCCCGCTTGGCCTGTCTACCCCTCCTTTGGCAGCAAATTTTTCTGCTGCCTCGTTTGACTTTAGTGGGGTGTCAATTTTTTTTGGCAACCCCGCTATCGCCACTGTCCCTCATCCATCGTCCCAACAAAATGCACTCATTCAATCGCATCGTCTTTTGACTCGATCTCTCCATGGTTCGTTGTGCTAATCATGCTTCAATCAATAGGAAGCCGCCGAACTCGGCAAGGGTTCCTTCAAGTATGCGTGGGTTCTTGACAAGCTCAAGGCCGAGCGTGAGCGTGGTATCACCATCGACATTGCCCTCTGGAAGTTCGAGACTCCCAAGTACTATGTCACCGT

		CATTGGTATGTTATTCCTGGCTCTTGACATGTCGAAATCATCATTCTAACGTACCAATACAGACG<br>CTCCCGGCCACCGTGAT
243R6	ACCATACCAAACTGTTGCCTCGGCGGGGTCACGCCCCGGGTGCGTCGCAGCCCCGGAACCA<br>GGCGCCCGCCGGAGGGACCAACCAAACTCTTTCTGTAGTCCCCTCGCGGACGTTATTTCTTAC<br>AGCTCTGAGCAAAAATTCAAAATGAATCAAAACTTTCAACAACGGATCTCTTGGTTCTGGCAT<br>CGATGAAGAACGCAGCGAAATGCGATAAGTAATGTGAATTGCAGAATTCAGTGAATCATCGAA<br>TCTTTGAACGCACATTGCGCCCGCCAGTATTCTGGCGGGCATGCCTGTCCGAGCGTCATTTCA<br>ACCCTCGAACCCCTCCGGGGGGTCGGCGTTGGGGATCGGGAACCCCTAAGACGGGATCCCG<br>GCCCCGAAATACAGTGGCGGTCTCGCCGCAGCCTCTCCTGCGCAGTAGTTTGCACAACTCGC<br>ACCGGGAGCGCGGCGCGTCCACGTCCGTAAAACACCCAACTTCTGAAATGTTGACCTCGGAT<br>CAGGTAGGAATACCCGCTGAACTTAAGCATATCAATAAGCGGAGGA	CTCATTCTTCTCGTCATCGGCACTCTCTATCTGCCGTTGACGCTGATGCGTATGACATCTTGCTG<br>ACCATAATCGTCTAGGGGTTCGTATTTTTCCATCAGACAGCTTTCGACCGACTTCAATACTGAC<br>TTGCTACAACAGCCACGTCGACTCCGGCAAGTCTACCACCGTGAGTAACTCTCAATTCCTCGA<br>GCCCTGCTGCCATTGACTCTGTCGGTCGGCGCGGGGTATCTTGATCTTGAACGCATCCAGCTA<br>ACATTTTCCCAATAGACTGGTCACTTGATCTACCAGTGCGGTGGTATTGACAAGCGTACCATCG<br>AGAAGTTCGAGAAGGTAAGCTCATTTCACTACTTTTCCCACCACGCTTGGCACAATCGTGTCC<br>GACAATTCTGTTCTCAGTCTTGTCTGTTTTCCTCGCAGCGTCACACCCCGCTTGGCCTGTCTAC<br>CCCTCCTTTGGCAGCAAATTTTTCTGCTGCCTCGTTTGACTTTAGTGGGGTGTCAATTTTTTTT<br>GGCAACCCCGCTATCGCCACTGTCCCTCATCCATCGTCCCAACAAAATGCACTCATTCAATCGC<br>ATCGTCTTTTGACTCGATCTCTCCATGGTTCGTTGTGCTAATCATGCTTCAATCAATAGGAAGCC<br>GCCGAACTCGGCAAGGGTTCCTTCAAGTATGCGTGGGTTCTTGACAAGCTCAAGGCCGAGC<br>GTGAGCGTGGTATCACCATCGACATTGCCCTCTGGAAGTTCGAGACTCCCAAGTACTATGTCA<br>CCGTCATTGGTATGTTATTCCTGGCTCTTGACATGTCGAAATCATCATTCTAACGTACCAATACA<br>GACGCTCCCGGCCACCGTGATTTCATCAAGAACATGAT
231K16	CTGTTGCCTCGGCGGGGTCACGCCCCGGGTGCGTCGCAGCCCCGGAACCAGGCGCCCGCCG<br>GAGGGACCAACCAAACTCTTTCTGTAGTCCCCTCGCGGACGTTATTTCTTACAGCTCTGAGCA<br>AAAATTCAAAATGAATCAAAACTTTCAACAACGGATCTCTTGGTTCTGGCATCGATGAAGAAC<br>GCAGCGAAATGCGATAAGTAATGTGAATTGCAGAATTCAGTGAATCATCGAATCTTTGAACGC<br>ACATTGCGCCCGCCAGTATTCTGGCGGGCATGCCTGTCCGAGCGTCATTTCAACCCTCGAACC<br>CCTCCGGGGGTCCGGCGTTGGGGATCGGGAACCCCTAAGACGGGATCCCGGCCCCGAAATA<br>CAGTGGCGGTCTCGCCGCAGCCTCTCCTGCGCAGTAGTTTGCACAACTCGCACCGGGAGCGC<br>GGCGCGTCCACGTCCGTAAAACACCCAACTTCTGAAATGTTGACCTCGGATCAGGTAGGAATA<br>CCCGCTGAACTTAAGCA	TCTATCTGCCGTTGACGCTGATGCGTATGACATCTTGCTGACCATAATCGTCTAGGGGTTCGTA<br>TTTTTCCATCAGACAGCTTTCGACCGACTTCAATACTGACTTGCTACAACAGCCACGTCGACTC<br>CGGCAAGTCTACCACCGTGAGTAACTCCCAATTCCTCGAGCCCTGCTGCCATTGACTCTGTCG<br>GTCGGCGCGGGGTATCTTGACCTTGAACGCATCCAGCTAACATTTTCCCAATAGACTGGTCAC<br>TTGATCTACCAGTGCGGTGGTATTGACAAGCGTACCATCGAGAAGTTCGAGAAGGTAAGCTC<br>ATTTCACTACTTTTTCTACCACGCTTGGCACAATTGTGCCCGACAATTCTGTTCTCAGTCTTGTC<br>TGTTTCCCTCGCAGCGTCACACCCCGCTTGGCCTGTCTACCCCTCCTTTGGCAGCAAATTTTTC<br>TGCTGCCTCGTTTGACTTTAGTGGGGTGTCAATTTTTTTTGGCAACCCCCGCTATCGCCACTGT<br>CCCTCATCAATCGTCCCAACAAAATGCACTCATTCAATCGCATCGTCTTTTGACTCGATTTCTCT<br>GTGGTTCGTTGTGCTAATCATGCTTCAATCAATAGGAAGCCGCCGAACTCGGCAAGGGTTCCT<br>TCAAGTATGCGTGGGTTCTTGACAAGCTCAAGGCCGAGCGTGAGCGTGGTATCACCATCGAC<br>ATTGCCCTCTGGAAGTTCGAGACTCCCAAGTACTATGTCACCGTCATTGGTATGTTATTCCTGG<br>CTCTTGACATGTCGAAATCATCATTCTAACGTGCCAATACAGACGCTCCCGG
134D16	CTCGGCGGGGTCACGCCCCGGGTGCGTCGCAGCCCCGGAACCAGGCGCCCGCCGGAGGGA<br>CCAACCAAACTCTTTCTGTAGTCCCCTCGCGGACGTTATTTCTTACAGCTCTGAGCAAAAATTC<br>AAAATGAATCAAAACTTTCAACAACGGATCTCTTGGTTCTGGCATCGATGAAGAACGCAGCG<br>AAATGCGATAAGTAATGTGAATTGCAGAATTCAGTGAATCATCGAATCTTTGAACGCACATTGC<br>GCCCGCCAGTATTCTGGCGGGCATGCCTGTCCGAGCGTCATTTCAACCCTCGAACCCCTCCGG<br>GGGGTCGGCGTTGGGGATCGGGAACCCCTAAGACGGGATCCCGGCCCCGAAATACAGTGGC<br>GGTCTCGCCGCAGCCTCTCCTGCGCAGTAGTTTGCACAACTCGCACCGGGAGCGCGGCGCGT<br>CCACGTCCGTAAAACACCCAACTTCTGAAATGTTGACCTCGGATCAGGTAGGAATACCCGCTG<br>AACTTAAGCATATCAATAAGCGGAGGAA	GTTTGCAATCCTCATTCTTGTCATCGGCACTCTCTATCTGCCGTTGACGCTGATGCGTATGACAT<br>CTTGCTGACCATAATCGTCTAGGGGTTCGTATTTTTCCATCAGACAGCTTTCGACCGACTTCAA<br>TACTGACTTGCTACAACAGCCACGTCGACTCCGGCAAGTCTACCACCGTGAGTAACTCCCAAT<br>TCCTCGAGCCCTGCTGCCATTGACTCTGTCGGTCGGCGCGGGGTATCTTGACCTTGAACGCAT<br>CCAGCTAACATTTTCCCAATAGACTGGTCACTTGATCTACCAGTGCGGTGGTATTGACAAGCG<br>TACCATCGAGAAGTTCGAGAAGGTAAGCTCATTTCACTACTTTTTCTACCACGCTTGGCACAA<br>TTGTGCCCGACAATTCTGTTCTCAGTCTTGTCTGTTTCCCTCGCAGCGTCACACCCCGCTTGGC<br>CTGTCTACCCCTCCTTTGGCAGCAAATTTTTCTGCTGCCTCGTTTGACTTTAGTGGGGTGTCAA<br>TTTTTTTTGGCAACCCCCGCTATCGCCACTGTCCCTCATCAATCGTCCCAACAAAATGCACTCA<br>TTCAATCGCATCGTCTTTTGACTCGATTTCTCTGTGGTTCGTTGTGCTAATCATGCTTCAATCAA<br>TAGGAAGCCGCCGAACTCGGCAAGGGTTCCTTCAAGTATGCGTGGGTTCTTGACAAGCTCAA<br>GGCCGAGCGTGAGCGTGGTATCACCATCGACATTGCCCTCTGGAAGTTCGAGACTCCCAAGT<br>ACTATGTCACCGTCATTGGTATGTTATTCCTGGCTCTTGACATGTCGAAATCATCATTCTAACGT<br>GCCAATACAGACGCTCCCGGCCACCGTGAT
141F9	ACTGTTGCCTCGGCGGGGTCACGCCCCGGGTGCGTCGCAGCCCCGGAACCAGGCGCCCGCC	TTCTATCTGCCGTTGACGCTGATGCGTATGACATCTTGCTGACCATAATCGTCTAGGGGTTCGT

	GGAGGGACCAACCAAACTCTTTCTGTAGTCCCCTCGCGGACGTTATTTCTTACAGCTCTGAGC<br>AAAAATTCAAAATGAATCAAAACTTTCAACAACGGATCTCTTGGTTCTGGCATCGATGAAGAA<br>CGCAGCGAAATGCGATAAGTAATGTGAATTGCAGAATTCAGTGAATCATCGAATCTTTGAACG<br>CACATTGCGCCCGCCAGTATTCTGGCGGGCATGCCTGTCCGAGCGTCATTTCAACCCTCGAAC<br>CCCTCCGGGGGTCCGGCGTTGGGGATCGGGAACCCCTAAGACGGGATCCCGGCCCCGAAAT<br>ACAGTGGCGGTCTCGCCGCAGCCTCTCCTGCGCAGTAGTTTGCACAACTCGCACCGGGAGCG<br>CGGCGCGTCCACGTCCGTAAAACACCCAACTTCTGAAATGTTGACCTCGGATCAGGTAGGAAT<br>ACCCGCTGA	ATTTTTCCATCAGACAGCTTTCGACCGACTTCAATACTGACTTGCTACAACAGCCACGTCGACT<br>CCGGCAAGTCTACCACCGTGAGTAACTCTCAATTCCTCGAGCCCTGCTGCCATTGACTCTGTC<br>GGTCGGCGCGGGGTATCTTGATCTTGAACGCATCCAGCTAACATTTTCCCAATAGACTGGTCA<br>CTTGATCTACCAGTGCGGTGGTATTGACAAGCGTACCATCGAGAAGTTCGAGAAGGTAAGCT<br>CATTTCACTACTTTTCCCACCACGCTTGGCACAATCGTGTCCGACAATTCTGTTCTCAGTCTTG<br>TCTGTTTTCCTCGCAGCGTCACACCCCGCTTGGCCTGTCTACCCCTCCTTTGGCAGCAAATTTT<br>TCTGCTGCCTCGTTTGACTTTAGTGGGGTGTCAATTTTTTTTGGCAACCCCCGCTATCGCCACT<br>GTCCCTCATCCATCGTCCCAACAAAATGCACTCATTCAATCGCATCGTCTTTTGACTCGATCTCT<br>CCATGGTTCGTTGTGCTAATCATGCTTCAATCAATAGGAAGCCGCCGAACTCGGCAAGGGTTC<br>CTTCAAGTATGCGTGGGTTCTTGACAAGCTCAAGGCCGAGCGTGAGCGTGGTATCACCATCG<br>ACATTGCCCTCTGGAAGTTCGAGACTCCCAAGTACTATGTCACCGTCATTGGTATGTTATTCCT<br>GGCTCTTGACATGTCGAAATCATCATTCTAACGTACCAATACAGACGCTCCCGGCCACCGTGAT<br>TTC
561A7	TTGCCTCGGCGGGGTCACGCCCCGGGTGCGTCGCAGCCCCGGAACCAGGCGCCCGCCGGA<br>GGGACCAACCAAACTCTTTCTGTAGTCCCCTCGCGGACGTTATTTCTTACAGCTCTGAGCAAA<br>AATTCAAAATGAATCAAAACTTTCAACAACGGATCTCTTGGTTCTGGCATCGATGAAGAACGC<br>AGCGAAATGCGATAAGTAATGTGAATTGCAGAATTCAGTGAATCATCGAATCTTTGAACGCAC<br>ATTGCGCCCGCCAGTATTCTGGCGGGCATGCCTGTCCGAGCGTCATTTCAACCCTCGAACCCC<br>TCCGGGGGGTCGGCGTTGGGGATCGGGAACCCCTAAGACGGGATCCCGGCCCCGAAATACA<br>GTGGCGGTCTCGCCGCAGCCTCTCCTGCGCAGTAGTTTGCACAACTCGCACCGGGAGCGCG<br>GCGCGTCCACGTCCGTAAAACACCCAACTTCTGAAATGTTGACCTCGGATCAGGTAGGAATAC<br>CCGCTGAACTTA	GCACTCTCTATCTGCCGTTGACGCTGATGCGTATGGCATCTTGCTGACCATAATCGTCTAGGGG<br>TTCGTATTTTTCCATCAGACAGCTTTCGACCGACTTCAATACTGACTTGCTACAACAGCCACGT<br>CGACTCCGGCAAGTCTACCACCGTGAGTAACTCCCAATTCCTCGAGCCCTGCTGCCATTGACT<br>CTGTCGGTCGGCGCGGGGTATCTTGATCTTGAACGCATCCAGCTAACATTTTCCCAATAGACT<br>GGTCACTTGATCTACCAGTGCGGTGGTATTGACAAGCGTACCATCGAGAAGTTCGAGAAGGT<br>AAGCTCATTTCACTACTTTTCCCACCACGCTTGGCACAATCGTGTCCGACAATTCTGTTCTCAG<br>TCTTGTCTGTTTTCCTCGCAGCGTCACACCCCGCTTGGCCTGTCTACCCCTCCTTTGGCAGCAA<br>ATTTTTCTGCTGCCTCGTTTGACTTTAGTGGGGTGTCAATTTTTTTTTTGGCAACCCCGCTATC<br>GCCACTGTCCCTCATCCATCGTCCCAACAAAATGCACTCATTCAATCGCATCGTCTTTTGACTC<br>GATCTCTCCATGGTTCGTTGTGCTAATCATGCTTCAATCAATAGGAAGCCGCCGAACTCGGCA<br>AGGGTTCCTTCAAGTATGCGTGGGTTCTTGACAAGCTCAAGGCCGAGCGTGAGCGTGGTATC<br>ACCATCGACATTGCCCTCTGGAAGTTCGAGACTCCCAAGTACTATGTCACCGTCATTGGTATGT<br>TATTCCTGGCTCTTGACATGTCGAAATCATCATTCTAACGTGCCAATACAGACGCTCCCGGCCA<br>CCGTGATTTCATCAAGAAC
625J11	GTTGCCTCGGCGGGGTCACGCCCCGGGTGCGTCGCAGCCCCGGAACCAGGCGCCCGCCGG<br>AGGGACCAACCAAACTCTTTCTGTAGTCCCCTCGCGGACGTTATTTCTTACAGCTCTGAGCAA<br>AAATTCAAAATGAATCAAAACTTTCAACAACGGATCTCTTGGTTCTGGCATCGATGAAGAACG<br>CAGCGAAATGCGATAAGTAATGTGAATTGCAGAATTCAGTGAATCATCGAATCTTTGAACGCA<br>CATTGCGCCCGCCAGTATTCTGGCGGGCATGCCTGTCCGAGCGTCATTTCAACCCTCGAACCC<br>CTCCGGGGGGTCGGCGTTGGGGATCGGGAACCCCTAAGACGGGATCCCGGCCCCGAAATAC<br>AGTGGCGGTCTCGCCGCAGCCTCTCCTGCGCAGTAGTTTGCACAACTCGCACCGGGAGCGC<br>GGCGCGTCCACGTCCGTAAAACACCCAACTTCTGAAATGTTGACCTCGGATCAGGTAGGAATA<br>CCCGCTGAACTTAAGCATAT	TCGGCACTCTCTATCTGCCGTTGACGCTGATGCGTATGACATCTTGCTGACCATAATCGTCTAG<br>GGGTTCGTATTTTTCCATCAGACAGCTTTCGACCGACTTCAATACTGACTTGCTACAACAGCC<br>ACGTCGACTCCGGCAAGTCTACCACCGTGAGTAACTCCCAATTCCTCGAGCCCTGCTGCCATT<br>GACTCTGTCGGTCGGCGCGGGGTATCTTGACCTTGAACGCATCCAGCTAACATTTTCCCAATA<br>GACTGGTCACTTGATCTACCAGTGCGGTGGTATTGACAAGCGTACCATCGAGAAGTTCGAGA<br>AGGTAAGCTCATTTCACTACTTTTTCTACCACGCTTGGCACAATTGTGCCCGACAATTCTGTTC<br>TCAGTCTTGTCTGTTTCCCTCGCAGCGTCACACCCCGCTTGGCCTGTCTACCCCTCCTTTGGCA<br>GCAAATTTTTCTGCTGCCTCGTTTGACTTTAGTGGGGTGTCAATTTTTTTTGGCAACCCCCGCT<br>ATCGCCACTGTCCCTCATCAATCGTCCCAACAAAATGCACTCATTCAATCGCATCGTCTTTTGAC<br>TCGATTTCTCTGTGGTTCGTTGTGCTAATCATGCTTCAATCAATAGGAAGCCGCCGAACTCGGC<br>AAGGGTTCCTTCAAGTATGCGTGGGTTCTTGACAAGCTCAAGGCCGAGCGTGAGCGTGGTAT<br>CACCATCGACATTGCCCTCTGGAAGTTCGAGACTCCCAAGTACTATGTCACCGTCATTGGTATG<br>TTATTCCTGGCTCTTGACATGTCGAAATCATCATTCTAACGTGCCAATACAGACGCTCCCGGCC<br>ACCGTGAT
651A8	ACGCCCCGGGTGCGTCGCAGCCCCGGAACCAGGCGCCCGCCGGAGGGACCAACCAAACTC<br>TTTCTGTAGTCCCCTCGCGGACGTTATTTCTTACAGCTCTGAGCAAAAATTCAAAATGAATCAA<br>AACTTTCAACAACGGATCTCTTGGTTCTGGCATCGATGAAGAACGCAGCGAAATGCGATAAGT	TGATGCGTATGACATCTTGCTGACCATAATCGTCTAGGGGTTCGTATTTTTCCATCAGACAGCT<br>TTCGACCGACTTCAATACTGACTTGCTACAACAGCCACGTCGACTCCGGCAAGTCTACCACCG<br>TGAGTAACTCTCAATTCCTCGAGCCCTGCTGCCATTGACTCTGTCGGTCGGCGCGGGGTATCT

	AATGTGAATTGCAGAATTCAGTGAATCATCGAATCTTTGAACGCACATTGCGCCCGCCAGTATT<br>CTGGCGGGCATGCCTGTCCGAGCGTCATTTCAACCCTCGAACCCCTCCGGGGGGTCGGCGTT<br>GGGGATCGGGAACCCCTAAGACGGGATCCCGGCCCCGAAATACAGTGGCGGTCTCGCCGCA<br>GCCTCTCCTGCGCAGTAGTTTGCACAACTCGCACCGGGAGCGCGGCGCGTCCACGTCCGTAA<br>AACACCCAACTTCTGAAATGTTGACCTCGGATCAGGTAGGAATACCCGCTGAACTTAAGCATA<br>TCAATAAGCGGAGG	TGATCTTGAACGCATCCAGCTAACATTTTCCCAATAGACTGGTCACTTGATCTACCAGTGCGGT<br>GGTATTGACAAGCGTACCATCGAGAAGTTCGAGAAGGTAAGCTCATTTCACTACTTTTCCCAC<br>CACGCTTGGCACAATCGTGTCCGACAATTCTGTTCTCAGTCTTGTCTGTTTTCCTCGCAGCGTC<br>ACACCCCGCTTGGCCTGTCTACCCCTCCTTTGGCAGCAAATTTTTCTGCTGCCTCGTTTGACTT<br>TAGTGGGGTGTCAATTTTTTTTTGGCAACCCCGCTATCGCCACTGTCCCTCATCCATCGTCCCA<br>ACAAAATGCACTCATTCAATCGCATCGTCTTTTGACTCGATCTCTCCATGGTTCGTTGTGCTAAT<br>CATGCTTCAATCAATAGGAAGCCGCCGAACTCGGCAAGGGTTCCTTCAAGTATGCGTGGGTT<br>CTTGACAAGCTCAAGGCCGAGCGTGAGCGTGGTATCACCATCGACATTGCCCTCTGGAAGTT<br>CGAGACTCCCAAGTACTATGTCACCGTCATTGGTATGTTATTCCTGGCTCTTGACATGTCGAAA<br>TCATCATTCTAACGTACCAATACAGACGCTCCCGGCCACCGTGATTTCATCAAGAACATGATCA<br>CT
215E14	GTGAACGTTACCAAACTGTTGCCTCGGCGGGATCTCTGCCCCGGGTGCGTCGCAGCCCCGGA<br>CCAAGGCGCCCGCCGGAGGACCAACCTAAAACTCTTATTGTATACCCCCTCGCGGGTTTTTTT<br>TATAATCTGAGCCTTCTCGGCGCCTCTCGTAGGCGTTTCGAAAATGAATCAAAACTTTCAACAA<br>CGGATCTCTTGGTTCTGGCATCGATGAAGAACGCAGCGAAATGCGATAAGTAATGTGAATTGC<br>AGAATTCAGTGAATCATCGAATCTTTGAACGCACATTGCGCCCGCCAGTATTCTGGCGGGCAT<br>GCCTGTCCGAGCGTCATTTCAACCCTCGAACCCCTCCGGGGGGTCGGCGTTGGGGATCGGCC<br>CTCCCTTAGCGGGTGGCCGTCTCCGAAATACAGTGGCGGTCTCGCCGCAGCCTCTCCTGCGC<br>AGTAGTTTGCACACTCGCATCGGGAGCGCGGCGCGTCCACAGCCGTTAAACACCCAACTTC	TACGTATCATCCTTTCTTCATGTCGGCATCATTCGCCGCTCTGATTCTCAAACACTTGTGCTAAC<br>CACCATCTTCTAGGGGTGCGTATTCCATCAATCATCTTGAATGAGATCGATCGAACACAGTACT<br>GACTTGCTACAACAGCCACGTCGACTCCGGAAAGTCGACCACCGTGAGTTGCACCCTCTTCT<br>CCTGCTCCGATATCAAACGTCGTTTGATACGGGACATCTACTCTTTGAACACAGGGCTAATCAT<br>TTATCATACAGACCGGTCACTTGATCTACCAGTGCGGTGGTATCGACCGTCGTACCATCGAGA<br>AGTTCGAGAAGGTAAGCTTCAACTCATTTTCGCCTCGATTCTCCCTCCACATTTAATTGTGCCC<br>GATAATTCTGCAGAGAATTTTCGTGTCGACAATTTTTCATCACCCCGATTTGCATTACCCCTCCT<br>TTGCAGCGACGCAAATTTTTTTGGCTGTCGTTTGGTTTTAGTGGGGTTTCTCGTGCACCCCAC<br>TAGGTCACTGCTTTTTTTCTGCTTCGCTCTTACTGCCCAGCCATCATTCAACGTGCTCTGCGTCT<br>CATCACTTTCAGCGATGCTAACCACTTTTCCATCAATAGGAAGCCGCCGAACTCGGCAAGGGT<br>TCCTTCAAGTACGCTTGGGTTCTTGACAAGCTCAAGGCCGAGCGTGAGCGTGGTATCACCAT<br>CGACATTGCTCTGTGGAAGTTCGAGACTCCCAAGTACTATGTCACCGTCATTGGTATGTTCTTT<br>CCATCAACTTCACACAGCGATTACAAGCCAGTGCTAACAAGCAATTCACAGACGCTCCCGGC<br>CACCGTGATT
223H3	CCCAATGTGAACGTTACCAAACTGTTGCCTCGGCGGGATCTCTGCCCCGGGTGCGTCGCAGC<br>CCCGGACCAAGGCGCCCGCCGGAGGACCAACCTAAAACTCTTATTGTATACCCCCTCGCGGG<br>TTTTTTTTATAATCTGAGCCTTCTCGGCGCCTCTCGTAGGCGTTTCGAAAATGAATCAAAACTT<br>TCAACAACGGATCTCTTGGTTCTGGCATCGATGAAGAACGCAGCGAAATGCGATAAGTAATGT<br>GAATTGCAGAATTCAGTGAATCATCGAATCTTTGAACGCACATTGCGCCCGCCAGTATTCTGG<br>CGGGCATGCCTGTCCGAGCGTCATTTCAACCCTCGAACCCCTCCGGGGGGTCGGCGTTGGG<br>GATCGGCCCTCCCTTAGCGGGTGGCCGTCTCCGAAATACAGTGGCGGTCTCGCCGCAGCCTC<br>TCCTGCGCAGTAGTTTGCACACTCGCATCGGGAGCGCGGCGCGTCCACAGCCGTTAAACACC<br>CAACTTCTGAAATGTTGACCTCGGATCAGGTAGGAATACCC	TCATCCTTTCTTCATGTCGGCATCATTCGCCGCTCTGATTCTCAAACACTTGTGCTAACCACCAT<br>CTTCTAGGGGTGCGTATTCCATCAATCATCTTGAATGAGATCGATCGAACACAGTACTGACTTG<br>CTACAACAGCCACGTCGACTCCGGAAAGTCGACCACCGTGAGTTGCACCCTCTTCTCCTGCTC<br>CGATATCAAACGTCGTTTGATACGGGACATCTACTCTTTGAACACAGGGCTAATCATTTATCATA<br>CAGACCGGTCACTTGATCTACCAGTGCGGTGGTATCGACCGTCGTACCATCGAGAAGTTCGA<br>GAAGGTAAGCTTCAACTCATTTTCGCCTCGATTCTCCCTCCACATTTAATTGTGCCCGATAATTC<br>TGCAGAGAATTTTCGTGTCGACAATTTTTCATCACCCCGATTTGCATTACCCCTCCTTTGCAGC<br>GACGCAAATTTTTTTGGCTGTCGTTTGGTTTTAGTGGGGTTTCTCGTGCACCCCACTAGGTCA<br>CTGCTTTTTTTCTGCTTCGCTCTTACTGCCCAGCCATCATTCAACGTGCTCTGCGTCTCATCACT<br>TTCAGCGATGCTAACCACTTTTCCATCAATAGGAAGCCGCCGAACTCGGCAAGGGTTCCTTCA<br>AGTACGCTTGGGTTCTTGACAAGCTCAAGGCCGAGCGTGAGCGTGGTATCACCATCGACATT<br>GCTCTGTGGAAGTTCGAGACTCCCAAGTACTATGTCACCGTCATTGGTATGTTCTTTCCATCAA<br>CTTCACACAGCGATTACAAGCCAGTGCTAACAAGCAATTCACAGACGCTCCCGGCCAC
223H16	CCAAACTGTTGCCTCGGCGGGATCTCTGCCCCGGGTGCGTCGCAGCCCCGGACCAAGGCGC<br>CCGCCGGAGGACCAACCAAAACTCTTATTGTATACCCCCTCGCGGGTTTTTTTATAATCTGAG<br>CCTTCTCGGCGCCTCTCGTAGGCGTTTCGAAAATGAATCAAAACTTTCAACAACGGATCTCTT<br>GGTTCTGGCATCGATGAAGAACGCAGCGAAATGCGATAAGTAATGTGAATTGCAGAATTCAG<br>TGAATCATCGAATCTTTGAACGCACATTGCGCCCGCCAGTATTCTGGCGGGCATGCCTGTCCG<br>AGCGTCATTTCAACCCTCGAACCCCTCCGGGGGGTCGGCGTTGGGGATCGGCCCTGCCTTGG	TGTGCTAATCAACGCCTTCTAGGGGTGCGTATTCCATCAATCATCTTGAATGAGATCGATCGAA<br>CATAATACTGACTTGCTATAACAGCCACGTCGACTCCGGAAAGTCGACCACCGTAAGTTGCGC<br>CCTCTCTTGCTCCGATATCAAGCGTCGTTTGATGCGGTTCACCTATTGAACACAGGGCTAACCA<br>TGCATAATACAGACCGGTCACTTGATCTACCAGTGCGGTGGTATCGACCGTCGTACCATCGAG<br>AAGTTCGAGAAGGTAAGCTTCAACTGATTTTCGCCTCGATTCTTCCTCCTCCACATTCAATTGT<br>GCCCGACAATTCTGCAGAGAATTTTCGTGTCGACAATTTTTCATCACCCCGCTTTCCATTACCC

	CGGTGGCCGTCTCCGAAATACAGTGGCGGTCTCGCCGCAGCCTCTCCTGCGCAGTAGTTTGC<br>ACACTCGCATCGGGAGCGCGGCGCGTCCACAGCCGTTAAACACCCAACTTCTGAAATGTTGA<br>CCTCGGATCAGGTAGGAATACCCGCTGAACTTAAGCATATCAATAAGCGGAGGAA	CTCCTTTGCAGCGACGCAAATTTTTTTTGCTGTCGTTTGGTTTTTAGTGGGGTTCTCTGTGCA<br>ACCCCACTAGCTCCCTGCTTTTTCCTGCTTCACCTTCACTTCCTCGTCATCATTCAACGTGCTCT<br>GCGTCTTTGGTCATTCAGCGACGCTAACCACTTTTCCATCAATAGGAAGCCGCCGAACTCGGC<br>AAGGGTTCCTTCAAGTACGCTTGGGTTCTTGACAAGCTCAAGGCCGAGCGTGAGCGTGGTAT<br>CACCATTGACATTGCTCTGTGGAAGTTCGAGACTCCCAAGTACTATGTCACCGTCATTGGTAA<br>GTCTTCACTAAGTTCATGCTGCAATTGCGGACCAGTGCTAACAGGCAATTCACAGACGCTCCC<br>GGCCACCGTGATTTCATCAAGAACATGATCACTGGTAC
231K4	TGCCTCGGCGGGATCTCTGCCCCGGGTGCGTCGCAGCCCCGGACCAAGGCGCCCGCCGGAG<br>GACCAACCAAAACTCTTATTGTATACCCCCTCGCGGGTTTTTTTTATAATCTGAGCCTTCTCGGC<br>GCCTCTCGTAGGCGTTTCGAAAATGAATCAAAACTTTCAACAACGGATCTCTTGGTTCTGGCA<br>TCGATGAAGAACGCAGCGAAATGCGATAAGTAATGTGAATTGCAGAATTCAGTGAATCATCGA<br>ATCTTTGAACGCACATTGCGCCCGCCAGTATTCTGGCGGGCATGCCTGTCCGAGCGTCATTTC<br>AACCCTCGAACCCCTCCGGGGGGTCGGCGTTGGGGATCGGCCCTGCCTTGGCGGTGGCCGT<br>CTCCGAAATACAGTGGCGGTCTCGCCGCAGCCTCTCCTGCGCAGTAGTTTGCACACTCGCATC<br>GGGAGCGCGGCGCGTCCACAGCCGTTAAACACCCAACTTCTGAAATGTTGACCTCGGATCAG<br>GTAGGAATACCCGCTGAACTTAAGCATAT	CTGTTCCCTCACGTCGGCGTCATTCGCCGGTCTCATTCTCAAACACTTGTGCTAACCATTGCCT<br>TCTAGGGGTGCGTATTCCATCAATCATCTTGAATGAGATCGATCGAACATAATACTGACTTGCTA<br>TAACAGCCACGTCGACTCCGGAAAGTCGACCACCGTAAGTTGCGCCCTCTCTTGCTCCGATAT<br>CAAGCGTCGTTTGATGCGGTTCACCTATTGAACACAGGGCTAACCATGCATAATACAGACCGG<br>TCACTTGATCTACCAGTGCGGTGGTATCGACCGTCGTACCATCGAGAAGTTCGAGAAGGTAA<br>GCTTCAACTGATTTTCGCCTCGATTCTTCCTCCTCCACATTCAATTGTGCCCGACAATTCTGCA<br>GAGAATTTTCGTGTCGACAATTTTTCATCACCCCGCTTTCCATTACCCCTCCTTTGCAGCGACG<br>CAAATTTTTTTTGCTGTCGTTTGGTTTTTAGTGGGGTTCTCTGTGCAACCCCACTAGCTCCCTG<br>CTTTTTCCTGCTTCACCTTCACTTCCTCGTCATCATTCAACGTGCTCTGCGTCTTTGGTCATTCA<br>GCGACGCTAACCACTTTTCCATCAATAGGAAGCCGCCGAACTCGGCAAGGGTTCCTTCAAGT<br>ACGCTTGGGTTCTTGACAAGCTCAAGGCCGAGCGTGAGCGTGGTATCACCATTGACATTGCT<br>CTGTGGAAGTTCGAGACTCCCAAGTACTATGTCACCGTCATTGGTAAGTCTTCACTAAGTTCAT<br>GCTGCAATTGCGGACCAGTGCTAACAGGCAATTCACAGACGCTCCCGGCCACCGTGATT
311A2	GGGATCTCTGCCCCGGGTGCGTCGCAGCCCCGGACCAAGGCGCCCGCCGGAGGACCAACCT<br>AAAACTCTTATTGTATACCCCCTCGCGGGTTTTTTTTATAATCTGAGCCTTCTCGGCGCCTCTCG<br>TAGGCGTTTCGAAAATGAATCAAAACTTTCAACAACGGATCTCTTGGTTCTGGCATCGATGAA<br>GAACGCAGCGAAATGCGATAAGTAATGTGAATTGCAGAATTCAGTGAATCATCGAATCTTTGA<br>ACGCACATTGCGCCCGCCAGTATTCTGGCGGGCATGCCTGTCCGAGCGTCATTTCAACCCTCG<br>AACCCCTCCGGGGGGTCGGCGTTGGGGATCGGCCCTCCCTTAGCGGGTGGCCGTCTCCGAA<br>ATACAGTGGCGGTCTCGCCGCAGCCTCTCCTGCGCAGTAGTTTGCACACTCGCATCGGGAGC<br>GCGGCGCGTCCACAGCCGTTAAACACCCAACTTCTGAAATGTTGACCTCGGATCAGGTAG	GGATGCCTTCTAGGGGTGCGTATTCCATCAATCATCTTGAATGAGATCGATCGAACACAATACT<br>GACTTGCTATAACAGCCACGTCGACTCCGGAAAGTCGACCACCGTAAGTTGCGCCCTCTCTTG<br>CTCCGATATCAAACATCGTTTGATGCGGGACACCTATTCTTGAACACAGGGCTAACCATGCATA<br>ATACAGACCGGTCACTTGATCTACCAGTGCGGTGGTATCGACCGTCGTACCATCGAGAAGTTC<br>GAGAAGGTAAGCTTCAACTGATTTTCGCCTCGATTCTTCCTCTCTCCACATTCAATTGTGCCCG<br>ACAATTCTGCAGAGAATTTTCGTGTCGACAATTTTTCATCACCCCGCTTTCCATTACCCCTCCTT<br>TGCAGCGACGCAAATTTTTTTTGCTGTCGTTTGGTTTTTAGTGGGGTTCTCTGTGCAACCCCA<br>CTAGCTCCCTGCTTTTTCCTGCTTCACTCTCACTTCCTCGTCATCATTCAACGTGCTCTGCGTCT<br>TTGGTCATTCAGCGACGCTAACCACTTTTACATCAATAGGAAGCCGCCGAACTCGGCAAGGG<br>TTCCTTCAAGTACGCTTGGGTTCTTGACAAGCTCAAGGCCGAGCGTGAGCGTGGTATCACCA<br>TTGACATTGCTCTGTGGAAGTTCGAGACTCCCAAGTACTATGTCACCGTCATTGGTAAGTCTTC<br>ACTAAGTTCATGCTGCAATTGCGGACCAGTGCTAACAGGCAATTCACAGACGCTCCCGGCCA<br>CCGTGATTTCATCAAGAACATGATCACTGGTACTTCCCAGGCCGATTGCGCTA
333C1	GTGAACGTTACCAAACTGTTGCCTCGGCGGGATCTCTGCCCCGGGTGCGTCGCAGCCCCGGA<br>CCAAGGCGCCCGCCGGAGGACCAACCTAAAACTCTTATTGTATACCCCCTCGCGGGTTTTTTT<br>TATAATCTGAGCCTTCTCGGCGCCTCTCGTAGGCGTTTCGAAAATGAATCAAAACTTTCAACAA<br>CGGATCTCTTGGTTCTGGCATCGATGAAGAACGCAGCGAAATGCGATAAGTAATGTGAATTGC<br>AGAATTCAGTGAATCATCGAATCTTTGAACGCACATTGCGCCCGCCAGTATTCTGGCGGGCAT<br>GCCTGTCCGAGCGTCATTTCAACCCTCGAACCCCTCCGGGGGGTCGGCGTTGGGGATCGGCC<br>CTCCCTTAGCGGGTGGCCGTCTCCGAAATACAGTGGCGGTCTCGCCGCAGCCTCTCCTGCGC<br>AGTAGTTTGCACACTCGCATCGGGAGCGCGGCGCGTCCACAGCCGTTAAACACCCAACTTCT<br>GAAATGTTGACCTCGGATCAGGTAG	AAGGGGGTGCGTATTCCATCAATCATCTTGAATGAGATCAGATCGAACATAATACTGACTTGCT<br>ATAACAGCCACGTCGACTCCGGAAAGTCGACCACCGTAAGTTGCGCCCTCTCTTGCTCCGATA<br>TCAAGCGTCGTTTGATGCGGTTCACCTATTGAACACAGGGCTAACCATGCATAATACAGACCG<br>GTCACTTGATCTACCAGTGCGGTGGTATCGACCGTCGTACCATCGAGAAGTTCGAGAAGGTA<br>AGCTTCAACTGATTTTCGCCTCGATTCTTCCTCCTCCACATTCAATTGTGCCCGACAATTCTGCA<br>GAGAATTTTCGTGTCGACAATTTTTCATCACCCCGCTTTCCATTACCCCTCCTTTGCAGCGACG<br>CAAATTTTTTTTGCTGTCGTTTGGTTTTTAGTGGGGTTCTCTGTGCAACCCCACTAGCTCCCTG<br>CTTTTTCCTGCTTCACCTTCACTTCCTCGTCATCATTCAACGTGCTCTGCGTCTTTGGTCATTCA<br>GCGACGCTAACCACTTTTCCATCAATAGGAAGCCGCCGAACTCGGCAAGGGTTCCTTCAAGT<br>ACGCTTGGGTTCTTGACAAGCTCAAGGCCGAGCGTGAGCGTGGTATCACCATTGACATTGCT<br>CTGTGGAAGTTCGAGACTCCCAAGTACTATGTCACCGTCATTGGTAAGTCTTCACTAAGTTCAT

		GCTGCAATTGCGGACCAGTGCTAACAGGCAATTCACAGACGCTCCCGGCCACCGTGATTTCA
		TCAAGA
333C17	ACTGTTGCCTACGGCGGGGTACACGCCCCGGGTGCGTACGCAGCCCCGGAACCAGGCGCCC	AGCCGTTGACGCTGATGCGTATGACATCTTGCTGACCATAATCGTCTAGGGGTTCGTATTTTTC
	GCCGGAGGGACCAACCAAACTCTTTCTGTGGTCCCCTCGCGGACGTTATTTCTTACAGCTCTG	CATCAGACAGCTTTCGACCGACTTCAATACTGACTTGCTACAACAGCCACGTCGACTCCGGCA
	AGCAAAAATTCAAAATGAATCAAAACTTTCAACAACGGATCTCTTGGTTCTGGCATCGATGAA	AGTCTACCACCGTGAGTAACTCCCAATTCCTCGAGCCCTGCTGCCATTGACTCTGTCGGTCGG
	GAACGCAGCGAAATGCGATAAGTAATGTGAATTGCAGAATTCAGTGAATCATCGAATCTTTGA	CGCGGGGTATCTTGATCTTGAACGCATCCAGCTAACATTTTCCCAATAGACTGGTCACTTGATC
	ACGCACATTGCGCCCGCCAGTATTCTGGCGGGCATGCCTGTCCGAGCGTCATTTCAACCCTCG	TACCAGTGCGGTGGTATTGACAAGCGTACCATCGAGAAGTTCGAGAAGGTAAGCTCATTTCA
	AACCCCTCCGGGGGGTCGGCGTTGGGGATCGGGAACCCCTAAGACGGGATCCCGGCCCCGA	CTACTTTTTCCACCACGCTTGGCACAATCGTGTCCGACAATTCTGTTCTCAGTCTTGTCTGTTTT
	AATACAGTGGCGGTCTCGCCGCAGCCTCTCCTGCGCAGTAGTTTGCACAACTCGCACCGGGA	CCTCGCAGCGTCACACCCCGCTTGGCCTGTCTACCCCTCCTTTGGCAGCAAATTTTTCTGCTG
	GCGCGGCGCGTCCACGTCCGTAAAACACCCAACTTCTGAAATGTTGACCTCGGATCAGGTAG	CCTCGTTTGACTTTAGTGGGGTGTCAATTTTTTTTTGCAACCCCCGCTATCGCCACTGTCCCTC
	GAATACCCGCTGAACTTAAGCATA	ATCCATCGTCCCAACAAAATGCACTCATTCAATCGCATCGTCTTTTGACTCGATCTCTCCATGGT
		TCGTTGTGCTAATCATGCTTCAATCAATAGGAAGCCGCCGAACTCGGCAAGGGTTCCTTCAAG
		TATGCGTGGGTTCTTGACAAGCTCAAGGCCGAGCGTGAGCGTGGTATCACCATCGACATTGC
		CCTCTGGAAGTTCGAGACTCCCAAGTACTATGTCACCGTCATTGGTATGTTATTCCTGGCTCTT
		GACATGTCGAAATCATCATTCTAATGTGCCAATAC
343I8	TTGCCTCGGCGGGATCTCTGCCCCGGGTGCGTCGCAGCCCCGGACCAAGGCGCCCGCCGGA	TGGTCGTTATCGTACGTACAATCCTGTTCCCTCACGTCGGCGTCATTCGCCGGTCTCATTCTCA
	GGACCAACCAAAACTCTTATTGTATACCCCCTCGCGGGTTTTTTTATAATCTGAGCCTTCTCG	AACACTTGTGCTAACCATTGCCTTCTAGGGGTGCGTATTCCATCAATCATCTTGAATGAGATCG
	GCGCCTCTCGTAGGCGTTTCGAAAATGAATCAAAACTTTCAACAACGGATCTCTTGGTTCTGG	ATCGAACATAATACTGACTTGCTATAACAGCCACGTCGACTCCGGAAAGTCGACCACCGTAAG
	CATCGATGAAGAACGCAGCGAAATGCGATAAGTAATGTGAATTGCAGAATTCAGTGAATCATC	TTGCGCCCTCTCTTGCTCCGATATCAAGCGTCGTTTGATGCGGTTCACCTATTGAACACAGGG
	GAATCTTTGAACGCACATTGCGCCCGCCAGTATTCTGGCGGGCATGCCTGTCCGAGCGTCATT	CTAACCATGCATAATACAGACCGGTCACTTGATCTACCAGTGCGGTGGTATCGACCGTCGTACC
	TCAACCCTCGAACCCCTCCGGGGGGTCGGCGTTGGGGATCGGCCCTGCCTTGGCGGTGGCC	ATCGAGAAGTTCGAGAAGGTAAGCTTCAACTGATTTTCGCCTCGATTCTTCCTCCTCCACATTC
	GTCTCCGAAATACAGTGGCGGTCTCGCCGCAGCCTCTCCTGCGCAGTAGTTTGCACACTCGCA	AATTGTGCCCGACAATTCTGCAGAGAATTTTCGTGTCGACAATTTTTCATCACCCCGCTTTCCA
	TCGGGAGCGCGGCGCGTCCACAGCCGTTAAACACCCAACTTCTGAAATGTTGACCTCGGATC	TTACCCCTCCTTTGCAGCGACGCAAATTTTTTTTGCTGTCGTTTGGTTTTTAGTGGGGTTCTCT
	AGGTAGGAATACCCGCTGAACTTAAGCAT	GTGCAACCCCACTAGCTCCCTGCTTTTTCCTGCTTCACCTTCACTTCCTCGTCATCATTCAACGT
		GCTCTGCGTCTTTGGTCATTCAGCGACGCTAACCACTTTTCCATCAATAGGAAGCCGCCGAAC
		TCGGCAAGGGTTCCTTCAAGTACGCTTGGGTTCTTGACAAGCTCAAGGCCGAGCGTGAGCG
		TGGTATCACCATTGACATTGCTCTGTGGAAGTTCGAGACTCCCAAGTACTATGTCACCGTCATT
		GGTAAGTCTTCACTAAGTTCATGCTGCAATTGCGGACCAGTGCTAACAGGCAATTCACAGAC
		GCTCCCGGCCACCGTGATTTCATCAAGAACATGATCACTGGTAC
461C1	AAACTGTTGCCTCGGCGGGATCTCTGCCCCGGGTGCGTCGCAGCCCCGGACCAAGGCGCCC	TTCATGTCGGCATCATTCGCCGCTCTGATTCTCAAACACTTGTGCTAACCACCATCTTCTAGGG
	GCCGGAGGACCAACCTAAAACTCTTATTGTATACCCCCTCGCGGGTTTTTTTATAATCTGAGC	GTGCGTATTCCATCAATCATCTTGAATGAGATCGATCGAACACAGTACTGACTTGCTACAACAG
	CTTCTCGGCGCCTCTCGTAGGCGTTTCGAAAATGAATCAAAACTTTCAACAACGGATCTCTTG	CCACGTCGACTCCGGAAAGTCGACCACCGTGAGTTGCACCCTCTTCTCCTGCTCCGATATCAA
	GTTCTGGCATCGATGAAGAACGCAGCGAAATGCGATAAGTAATGTGAATTGCAGAATTCAGT	ACGTCGTTTGATACGGGACATCTACTCTTTGAACACAGGGCTAATCATTTATCATACAGACCGG
	GAATCATCGAATCTTTGAACGCACATTGCGCCCGCCAGTATTCTGGCGGGCATGCCTGTCCGA	TCACTTGATCTACCAGTGCGGTGGTATCGACCGTCGTACCATCGAGAAGTTCGAGAAGGTAA
	GCGTCATTTCAACCCTCGAACCCCTCCGGGGGGTCGGCGTTGGGGATCGGCCCTCCCTTAGC	GCTTCAACTCATTTTCGCCTCGATTCTCCCTCCACATTTAATTGTGCCCGATAATTCTGCAGAGA
	GGGTGGCCGTCTCCGAAATACAGTGGCGGTCTCGCCGCAGCCTCTCCTGCGCAGTAGTTTGC	ATTTTCGTGTCGACAATTTTTCATCACCCCGATTTGCATTACCCCTCCTTTGCAGCGACGCAAA
	ACACTCGCATCGGGAGCGCGGCGCGTCCACAGCCGTTAAACACCCAACTTCTGAAATGTTGA	TTTTTTTGGCTGTCGTTTGGTTTTAGTGGGGTTTCTCGTGCACCCCACTAGGTCACTGCTTTTT
	CCTCGGATCAGGTAGGAATACCCGCTGAACTT	TTCTGCTTCGCTCTTACTGCCCAGCCATCATTCAACGTGCTCTGCGTCTCATCACTTTCAGCGAT
		GCTAACCACTTTTCCATCAATAGGAAGCCGCCGAACTCGGCAAGGGTTCCTTCAAGTACGCTT
		GGGTTCTTGACAAGCTCAAGGCCGAGCGTGAGCGTGGTATCACCATCGACATTGCTCTGTGG
		AAGTTCGAGACTCCCAAGTACTATGTCACCGTCATTGGTATGTTCTTTCCATCAACTTCACACA
		GCGATTACAAGCCAGTGCTAACAAGCAATTCACAGACGCTCCCGGCCACCGTGATTTCATCAA
		GAACATGATCACT
415E2	TTGCCTCGGCGGGATCTCTGCCCCGGGTGCGTCGCAGCCCCGGACCAAGGCGCCCGCCGGA	ACTTGTGCTAACCATTGCCTTCTAGGGGTGCGTATTCCATCAATCATCTTGAATGAGATCGATC

	GGACCAACCAAAACTCTTATTGTATACCCCTCGCGGGTTTTTTTATAATCTGAGCCTTCTCG<br>GCGCCTCTCGTAGGCGTTTCGAAAATGAATCAAAACTTTCAACAACGGATCTCTTGGTTCTGG<br>CATCGATGAAGAACGCAGCGAAATGCGATAAGTAATGTGAATTGCAGAATTCAGTGAATCATC<br>GAATCTTTGAACGCACATTGCGCCCGCCAGTATTCTGGCGGGCATGCCTGTCCGAGCGTCATT<br>TCAACCCTCGAACCCCTCCGGGGGGTCGGCGTTGGGGATCGGCCCTGCCTTGGCGGTGGCC<br>GTCTCCGAAATACAGTGGCGGTCTCGCCGCAGCCTCTCCTGCGCAGTAGTTTGCACACTCGCA<br>TCGGGAGCGCGGCGCGTCCACAGCCGTTAAACACCCAACTTCTGAAATGTTGACCTCGGATC<br>AGGTAGGAATACCCGCTGAACTTAAGCAT	GAACATAATACTGACTTGCTATAACAGCCACGTCGACTCCGGAAAGTCGACCACCGTAAGTTG<br>CGCCCTCTCTTGCTCCGATATCAAGCGTCGTTTGATGCGGTTCACCTATTGAACACAGGGCTA<br>ACCATGCATAATACAGACCGGTCACTTGATCTACCAGTGCGGTGGTATCGACCGTCGTACCATC<br>GAGAAGTTCGAGAAGGTAAGCTTCAACTGATTTTCGCCTCGATTCTTCCTCCTCCACATTCAA<br>TTGTGCCCGACAATTCTGCAGAGAATTTTCGTGTCGACAATTTTTCATCACCCCGCTTTCCATT<br>ACCCCTCCTTTGCAGCGACGCAAATTTTTTTGCTGTCGTTTGGTTTTTAGTGGGGTTCTCTGT<br>GCAACCCCACTAGCTCCCTGCTTTTTCCTGCTTCACCTTCACTTCCTCGTCATCATTCAACGTGC<br>TCTGCGTCTTTGGTCATTCAGCGACGCTAACCACTTTTCCATCAATAGGAAGCCGCCGAACTC<br>GGCAAGGGTTCCTTCAAGTACGCTTGGGTTCTTGACAAGCTCAAGGCCGAGCGTGAGCGTG<br>GTATCACCATTGACATTGCTCTGTGGAAGTTCGAGACTCCCAAGTACTATGTCACCGTCATTGG<br>TAAGTCTTCACTAAGTTCATGCTGCAATTGCGGACCAGTGCTAACAGGCAATTCACAGACGCT<br>C
452B7	CTCGGCGGGATCTCTGCCCCGGGTGCGTCGCAGCCCCGGACCAAGGCGCCCGCCGGAGGAC<br>CAACCTAAAACTCTTATTGTATACCCCTCGCGGGTTTTTTTATAATCTGAGCCTTCTCGGCGC<br>CTCTCGTAGGCGTTTCGAAAATGAATCAAAACTTTCAACAACGGATCTCTTGGTTCTGGCATC<br>GATGAAGAACGCAGCGAAATGCGATAAGTAATGTGAATTGCAGAATTCAGTGAATCATCGAAT<br>CTTTGAACGCACATTGCGCCCGCCAGTATTCTGGCGGGCATGCCTGTCCGAGCGTCATTTCAA<br>CCCTCGAACCCCTCCGGGGGGTCGGCGTTGGGGATCGGCCCTCCCTTAGCGGGTGGCCGTCT<br>CCGAAATACAGTGGCGGTCTCGCCGCAGCCTCTCCTGCGCAGTAGTTTGCACACTCGCATCG<br>GGAGCGCGGCGCGTCCACAGCCGTTAAACACCCAACTTCTGAAATGTTGACCTCGGATCAGG<br>TAGGAATA	GTCTCATTCTCAAAACACTTGTGCTAACCATTGCCTTCTAGGGGGTGCGTATTCCATCAATCAT<br>CTTGAATGAGATCGATCGAACATAATACTGACTTGCTATAACAGCCACGTCGACTCCGGAAAG<br>TCGACCACCGTAAGTTGCGCCCTCTCTTGCTCCGATATCAAGCGTCGTTTGATGCGGTTCACCT<br>ATTGAACACAGGGCTAACCATGCATAATACAGACCGGTCACTTGATCTACCAGTGCGGTGGTA<br>TCGACCGTCGTACCATCGAGAAGTTCGAGAAGGTAAGCTTCAACTGATTTTCGCCTCGATTCT<br>TCCTCCTCCACATTCAATTGTGCCCGACAATTCTGCAGAGAATTTTCGTGTCGACAATTTTTCA<br>TCACCCCGCTTTCCATTACCCCTCCTTTGCAGCGACGCAAATTTTTTTGCTGTCGTTTGGTTT<br>TTAGTGGGGTTCTCTGTGCAACCCCACTAGCTCCCTGCTTTTTCCTGCTTCACCTTCACTTCCT<br>CGTCATCATTCAACGTGCTCTGCGTCTTTGGTCATTCAGCGACGCTAACCACTTTTCCATCAAT<br>AGGAAGCCGCCGAACTCGGCAAGGGTTCCTTCAAGTACGCTTGGGTTCTTGACAAGCTCAA<br>GGCCGAGCGTGAGCGTGGTATCACCATTGACATTGCTCTGTGGAAGTTCGAGACTCCCAAGT<br>ACTATGTCACCGTCATTGGTAAGTCTTCACTAAGTTCATGCTGCAATTGCGGACCAGTGCTAAC<br>AGGCAATTCACAGACGCTCCCGGCC
552B7	CTGTTGCCTCGGCGGGATCTCTGCCCCGGGTGCGTCGCAGCCCCGGACCAAGGCGCCCGCC<br>GGAGGACCAACCAAAACTCTTATTGTATACCCCTCGCGGGTTTTTTTATAATCTGAGCCTTCT<br>CGGCGCCTCTCGTAGGCGTTTCGAAAATGAATCAAAACTTTCAACAACGGATCTCTTGGTTCT<br>GGCATCGATGAAGAACGCAGCGAAATGCGATAAGTAATGTGAATTGCAGAATTCAGTGAATC<br>ATCGAATCTTTGAACGCACATTGCGCCCGCCAGTATTCTGGCGGGCATGCCTGTCCGAGCGTC<br>ATTTCAACCCTCGAACCCCTCCGGGGGGTCGGCGTTGGGGATCGGCCCTGCCTTGGCGGTG<br>GCCGTCTCCGAAATACAGTGGCGGTCTCGCCGCAGCCTCTCCTGCGCAGTAGTTTGCACACTC<br>GCATCGGGAGCGCGGCGCGTCCACAGCCGTTAAACACCCAACTTCTGAAATGTTGACCTCGG<br>ATCAGGTAGGAATACCCGCTGAACTTAAGCA	AGCATCATTCGCCGGTCTGATTCTCAAACACTTGTGCTAACCATTGCCTTCTAGGGGTGCGTAT<br>TCCATCAATCATCTTGAATGAGATCGATCGAACACAATACTGACTTGCTATAACAGCCACGTCG<br>ACTCCGGAAAGTCGACCACCGTAAGTTGCGCCCTCTATTGCTCCGGTATCAAACGTCGTTTGA<br>TGCGGGACACCTATTCTTGAACACAGGGCTAACCATGCATAATACAGACCGGTCACTTGATCT<br>ACCAGTGCGGTGGTATCGACCGTCGTACCATCGAGAAGTTCGAGAAGGTAAGCTTCAACTGA<br>TTTTCGCCTCGATTCTTCCTCCTCCACATTCAATTGTGCCCGACAATTCTGCAGAGAATTTTCGT<br>GTCGACAATTTTTCATCACCCCGCTTTCCATTACCCCTCCTTTGCAGCGACGCAAATTTTTTT<br>GCTGTCGTTTGGTTTTTAGTGGGGTTCTCTGTGCAACCCCACTAGCTTCCTGCTTTTTCCTGCT<br>TCACTCTCACTTCCTCGTCATCATTCAACGTGCTCTGCGTCTTTGGTCATTCAGCGACGCTAAC<br>CACTTTTCCATCAATAGGAAGCCGCCGAACTCGGCAAGGGTTCCTTCAAGTACGCTTGGGTT<br>CTTGACAAGCTCAAGGCCGAGCGTGAGCGTGGTATCACCATTGACATTGCTCTGTGGAAGTT<br>CGAGACTCCCAAGTACTATGTCACCGTCATTGGTAAGTCTTCACTAAGTTCATGCTGCAATTGC<br>GGACCAGTGCTAACAGGCAATTCACAGACGCTCCCGGCCACCGTGAT
553C1	GTTACCAAACTGTTGCCTCGGCGGGATCTCTGCCCCGGGTGCGTCGCAGCCCCGGACCAAGG<br>CGCCCGCCGGAGGACCAACCTAAAACTCTTATTGTATACCCCTCGCGGGTTTTTTTATAATCT<br>GAGCCTTTCTCGGCGCCTCTCGTAGGCGTTTCGAAAATGAATCAAAACTTTCAACAACGGATC<br>TCTTGGTTCTGGCATCGATGAAGAACGCAGCGAAATGCGATAAGTAATGTGAATTGCAGAATT<br>CAGTGAATCATCGAATCTTTGAACGCACATTGCGCCCGCCAGTATTCTGGCGGGCATGCCTGT<br>CCGAGCGTCATTTCAACCCTCGAACCCCTCCGGGGGGTCGGCGTTGGGGATCGGCCCTCCCT	GATCTTCTAGGGGGTGCGTATTCCATCAATCATCTTGAATGAGATCGATCGAACACAGTACTGAC<br>TTGCTACAACAGCCACGTCGACTCCGGAAAGTCGACCACCGTGAGTTGCACCCTCTTCTCCTG<br>CTCCGATATCAAACGTCGTTTGATACGGGACATCTACTCTTTGAACACAGGGCTAATCATTTAT<br>CATACAGACCGGTCACTTGATCTACCAGTGCGGTGGTATCGACCGTCGTACCATCGAGAAGTT<br>CGAGAAGGTAAGCTTCAACTCATTTTCGCCTCGATTCTCCCTCCACATTTAATTGTGCCCGATA<br>ATTCTGCAGAGAATTTTCGTGTCGACAATTTTTCATCACCCCGATTTGCATTACCCCTCCTTTGC

	TAGCGGGTGGCCGTCTCCGAAATACAGTGGCGGTCTCGCCGCAGCCTCTCCTGCGCAGTAGT<br>TTGCACACTCGCATCGGGAGCGCGGCGCGTCCACAGCCGTTAAACACCCAACTTCTGAAATG<br>TTGACCTCGGATCAGGTAGGAATACCCGCTGAACTTAAG	AGCGACGCAAATTTTTTTGGCTGTCGTTTGGTTTTAGTGGGGTTTCTCGTGCACCCCACTAGG<br>TCACTGCTTTTTTTCTGCTTCGCTCTTACTGCCCAGCCATCATTCAACGTGCTCTGCGTCTCATC<br>ACTTTCAGCGATGCTAACCACTTTTCCATCAATAGGAAGCCGCCGAACTCGGCAAGGGTTCCT<br>TCAAGTACGCTTGGGTTCTTGACAAGCTCAAGGCCGAGCGTGAGCGTGGTATCACCATCGAC<br>ATTGCTCTGTGGAAGTTCGAGACTCCCAAGTACTATGTCACCGTCATTGGTATGTTCTTTCCAT<br>CAACTTCACACAGCGATTACAAGCCAGTGCTAACAAGCAATTCACAGACGCTCCCGG
563C3	ACGTTACCAAACTGTTGCCTCGGCGGGATCTCTGCCCCGGGTGCGTCGCAGCCCCGGACCAA<br>GGCGCCCGCCGGAGGACCAACCAAAACTCTTATTGTATACCCCCTCGCGGGTTTTTTTATAAT<br>CTGAGCCTTCTCGGCGCCTCTCGTAGGCGTTTCGAAAATGAATCAAAACTTTCAACAACGGAT<br>CTCTTGGTTCTGGCATCGATGAAGAACGCAGCGAAATGCGATAAGTAATGTGAATTGCAGAAT<br>TCAGTGAATCATCGAATCTTTGAACGCACATTGCGCCCGCCAGTATTCTGGCGGGCATGCCTG<br>TCCGAGCGTCATTTCAACCCTCGAACCCCTCCGGGGGGTCGGCGTTGGGGATC	TGCCTTCTAGGGGTGCGTATTCCATCAATCATCTTGAATGAGATCGATCGAACATAATACTGAC<br>TTGCTATAACAGCCACGTCGACTCCGGAAAGTCGACCACCGTAAGTTGCGCCCTCTCTTGCTC<br>CGATATCAAGCGTCGTTTGATGCGGTTCACCTATTGAACACAGGGCTAACCATGCATAATACA<br>GACCGGTCACTTGATCTACCAGTGCGGTGGTATCGACCGTCGTACCATCGAGAAGTTCGAGA<br>AGGTAAGCTTCAACTGATTTTCGCCTCGATTCTTCCTCCTCCACATTCAATTGTGCCCGACAAT<br>TCTGCAGAGAATTTTCGTGTCGACAATTTTTCATCACCCCGCTTTCCATTACCCCTCCTTTGCA<br>GCGACGCAAATTTTTTTTGCTGTCGTTTGGTTTTTAGTGGGGTTCTCTGTGCAACCCCACTAG<br>CTCCCTGCTTTTTCCTGCTTCACCTTCACTTCCTCGTCATCATTCAACGTGCTCTGCGTCTTTGG<br>TCATTCAGCGACGCTAACCACTTTTCCATCAATAGGAAGCCGCCGAACTCGGCAAGGGTTCCT<br>TCAAGTACGCTTGGGTTCTTGACAAGCTCAAGGCCGAGCGTGAGCGTGGTATCACCATTGAC<br>ATTGCTCTGTGGAAGTTCGAGACTCCCAAGTACTATGTCACCGTCATTGGTAAGTCTTCACTAA<br>GTTCATGCTGCAATTGCGGACCAGTGCTAACAGGCAATTCACAGACGCTCCCGGCCACCGTG<br>ATT
563C5	GAACGTTACCAAACTGTTGCCTCGGCGGGATCTCTGCCCCGGGTGCGTCGCAGCCCCGGACC<br>AAGGCGCCCGCCGGAGGACCAACCTAAAACTCTTATTGTATACCCCCTCGCGGGTTTTTTTAT<br>AATCTGAGCCTTCTCGGCGCCTCTCGTAGGCGTTTCGAAAATGAATCAAAACTTTCAACAACG<br>GATCTCTTGGTTCTGGCATCGATGAAGAACGCAGCGAAATGCGATAAGTAATGTGAATTGCAG<br>AATTCAGTGAATCATCGAATCTTTGAACGCACATTGCGCCCGCCAGTATTCTGGCGGGCATGC<br>CTGTCCGAGCGTCATTTCAACCCTCGAACCCCTCCGGGGGGTCGGCGTTGGGGATCGGCCCT<br>CCCTTAGCGGGTGGCCGTCTCCGAAATACAGTGGCGGTCTCGCCGCAGCCTCTCCTGCGCAG<br>TAGTTTGCACACTCGCATCGGGAGCGCGGCGCGTCCACAGCCGTTAAACACCCAACTTCTGA<br>AATGTTGACCTCGGATCAGGTA	CGGCGTCATTCGCCGGTCTCATTCTCAAACACTTGTGCTAACCATTGCCTTCTAGGGGTGCGTA<br>TTCCATCAATCATCTTGAATGAGATCGATCGAACATAATACTGACTTGCTATAACAGCCACGTC<br>GACTCCGGAAAGTCGACCACCGTAAGTTGCGCCCTCTCTTGCTCCGATATCAAGCGTCGTTTG<br>ATGCGGTTCACCTATTGAACACAGGGCTAACCATGCATAATACAGACCGGTCACTTGATCTACC<br>AGTGCGGTGGTATCGACCGTCGTACCATCGAGAAGTTCGAGAAGGTAAGCTTCAACTGATTT<br>TCGCCTCGATTCTTCCTCCTCCACATTCAATTGTGCCCGACAATTCTGCAGAGAATTTTCGTGT<br>CGACAATTTTTCATCACCCCGCTTTCCATTACCCCTCCTTTGCAGCGACGCAAATTTTTTTTGCT<br>GTCGTTTGGTTTTTAGTGGGGTTCTCTGTGCAACCCCACTAGCTCCCTGCTTTTTCCTGCTTCA<br>CCTTCACTTCCTCGTCATCATTCAACGTGCTCTGCGTCTTTGGTCATTCAGCGACGCTAACCAC<br>TTTTCCATCAATAGGAAGCCGCCGAACTCGGCAAGGGTTCCTTCAAGTACGCTTGGGTTCTT<br>GACAAGCTCAAGGCCGAGCGTGAGCGTGGTATCACCATTGACATTGCTCTGTGGAAGTTCGA<br>GACTCCCAAGTACTATGTCACCGTCATTGGTAAGTCTTCACTAAGTTCATGCTGCAATTGCGGA<br>CCAGTGCTAACAGGCAATTCACAGACGCTCCCGGCCACCGTGAT
611A17	GAACGTTACCAAACTGTTGCCTCGGCGGGATCTCTGCCCCGGGTGCGTCGCAGCCCCGGACC<br>AAGGCGCCCGCCGGAGGACCAACCAAAACTCTTATTGTATACCCCCTCGCGGGTTTTTTTAT<br>AATCTGAGCCTTCTCGGCGCCTCTCGTAGGCGTTTCGAAAATGAATCAAAACTTTCAACAACG<br>GATCTCTTGGTTCTGGCATCGATGAAGAACGCAGCGAAATGCGATAAGTAATGTGAATTGCAG<br>AATTCAGTGAATCATCGAATCTTTGAACGCACATTGCGCCCGCCAGTATTCTGGCGGGCATGC<br>CTGTCCGAGCGTCATTTCAACCCTCGAACCCCTCCGGGGGGTCGGCGTTGGGGATCGGCCCT<br>GCCTTGGCGGTGGCCGTCTCCGAAATACAGTGGCGGTCTCGCCGCAGCCTCTCCTGCGCAGT<br>AGTTTGCACACTCGCATCGGGAGCGCGGCGCGTCCACAGCCGTTAAACACCCAACTTCTGAA<br>ATGTTGACCTCGGATCAGGTAGGAATACCCGCTGAACTTAAGCATATCAATAAGCGGAGGAA	AACACTTGTGCTAACCATTGCCTTCTAGGGGTGCGTATTCCATCAATCATCTTGAATGAGATCG<br>ATCGAACATAATACTGACTTGCTATAACAGCCACGTCGACTCCGGAAAGTCGACCACCGTAAG<br>TTGCGCCCTCTCTTGCTCCGATATCAAGCGTCGTTTGATGCGGGTCACCTATTGAACACAGGG<br>CTAACCATGCATAATACAGACCGGTCACTTGATCTACCAGTGCGGTGGTATCGACCGTCGTACC<br>ATCGAGAAGTTCGAGAAGGTAAGCTTCAACTGATTTTCGCCTCGATTCTTCCTCCTCCACATTC<br>AATTGTGCCCGACAATTCTGCAGAGAATTTTCGTGTCGACAATTTTTCATCACCCCGCTTTCCA<br>TTACCCCTCCTTTGCAGCGACGCAAATTTTTTTTGCTGTCGTTTGGTTTTTAGTGGGGTTCTCT<br>GTGCAACCCCACTAGCTCCCTGCTTTTTCCTGCTTCACCTTCACTTCCTCGTCATCATTCAACGT<br>GCTCTGCGTCTTTGGTCATTCAGCGACGCTAACCACTTTTCCATCAATAGGAAGCCGCCGAAC<br>TCGGCAAGGGTTCCTTCAAGTACGCTTGGGTTCTTGACAAGCTCAAGGCCGAGCGTGAGCG<br>TGGTATCACCATTGACATTGCTCTGTGGAAGTTCGAGACTCCCAAGTACTATGTCACCGTCATT<br>GGTAAGTCTTCACTAAGTTCATGCTGCAATTGCGGACCAGTGCTAACAGGCAATTCACAGAC

		GCTCCCGGCCACCGTGATT
611A18	GTTGCCTCGGCGGGATCTCTGCCCCGGGTGCGTCGCAGCCCCGGACCAAGGCGCCCGCCGG<br>AGGACCAACCAAAACTCTTATTGTATACCCCCTCGCGGGTTTTTTTATAATCTGAGCCTTCTCG<br>GCGCCTCTCGTAGGCGTTTCGAAAATGAATCAAAACTTTCAACAACGGATCTCTTGGTTCTGG<br>CATCGATGAAGAACGCAGCGAAATGCGATAAGTAATGTGAATTGCAGAATTCAGTGAATCATC<br>GAATCTTTGAACGCACATTGCGCCCGCCAGTATTCTGGCGGGCATGCCTGTCCGAGCGTCATT<br>TCAACCCTCGAACCCCTCCGGGGGGTCGGCGTTGGGGATCGGCCCTGCCTTGGCGGTGGCC<br>GTCTCCGAAATACAGTGGCGGTCTCGCCGCAGCCTCTCCTGCGCAGTAGTTTGCACACTCGCA<br>TCGGGAGCGCGGCGCGTCCACAGCCGTTAAACACCCAACTTCTGAAATGTTGACCTCGGATC<br>AGGTAGGAATACCCGCTGAACTTAAGCAT	CGCCGGTCTCATTCTCAAACACTTGTGCTAACCATTGCCTTCTAGGGGTGCGTATTCCATCAAT<br>CATCTTGAATGAGATCGATCGAACATAATACTGACTTGCTATAACAGCCACGTCGACTCCGGAA<br>AGTCGACCACCGTAAGTTGCGCCCTCTCTTGCTCCGATATCAAGCGTCGTTTGATGCGGTTCA<br>CCTATTGAACACAGGGCTAACCATGCATAATACAGACCGGTCACTTGATCTACCAGTGCGGTG<br>GTATCGACCGTCGTACCATCGAGAAGTTCGAGAAGGTAAGCTTCAACTGATTTTCGCCTCGAT<br>TCTTCCTCCTCCACATTCAATTGTGCCCGACAATTCTGCAGAGAATTTTCGTGTCGACAATTTT<br>TCATCACCCCGCTTTCCATTACCCCTCCTTTGCAGCGACGCAAATTTTTTTTGCTGTCGTTTGG<br>TTTTTAGTGGGGTTCTCTGTGCAACCCCACTAGCTCCCTGCTTTTTCCTGCTTCACCTTCACTT<br>CCTCGTCATCATTCAACGTGCTCTGCGTCTTTGGTCATTCAGCGACGCTAACCACTTTTCCATC<br>AATAGGAAGCCGCCGAACTCGGCAAGGGTTCCTTCAAGTACGCTTGGGTTCTTGACAAGCTC<br>AAGGCCGAGCGTGAGCGTGGTATCACCATTGACATTGCTCTGTGGAAGTTCGAGACTCCCAA<br>GTACTATGTCACCGTCATTGGTAAGTCTTCACTAAGTTCATGCTGCAATTGCGGACCAGTGCTA<br>ACAGGCAATTCACAGACGCTCCCGGCCACCGTGATTTCATCAAGAACATGATC
612B8	TGTTGCCTCGGCGGGATCTCTGCCCCGGGGGCGTCCCACCCCCGGACCAAGGCGCCCGCCG<br>GAGGACCAACCAAAACTCTTATTGTATACCCCCTCGCGGGTTTTTTTATAATCTGAGCCTTCTC<br>GGCGCCTCTCGTAGGCGTTTCGAAAATGAATCAAAACTTTCAACAACGGATCTCTTGGTTCTG<br>GCATCGATGAAGAACGCAGCGAAATGCGATAAGTAATGTGAATTGCAGAATTCAGTGAATCAT<br>CGAATCTTTGAACGCACATTGCGCCCGCCAGTATTCTGGCGGGCATGCCTGTCCGAGCGTCAT<br>TTCAACCCTCGAACCCCTCCGGGGGGTCGGCGTTGGGGATCGGCCCTGCCTTGGCGGTGGC<br>CGTCTCCGAAATACAGTGGCGGTCTCGCCGCAGCCTCTCCTGCGCAGTAGTTTGCACACTCGC<br>ATCGGGAGCGCGGCGCGTCCACAGCCGTTAAACACCCAACTTCTGAAATGTTGACCTCGGAT<br>CAGGTAGGA	ATTGCCTTCTAGGGGGTGCGTATTCCATCAATCATCTTGAATGAGATCGATCGAACATAATACT<br>GACTTGCTATAACAGCCACGTCGACTCCGGAAAGTCGACCACCGTAAGTTGCGCCCTCTCTTG<br>CTCCGATATCAAGCGTCGTTTGATGCGGTTCACCTATTGAACACAGGGCTAACCATGCATAATA<br>CAGACCGGTCACTTGATCTACCAGTGCGGTGGTATCGACCGTCGTACCATCGAGAAGTTCGA<br>GAAGGTAAGCTTCAACTGATTTTCGCCTCGATTCTTCCTCCTCCACATTCAATTGTGCCCGACA<br>ATTCTGCAGAGAATTTTCGTGTCGACAATTTTTCATCACCCCGCTTTCCATTACCCCTCCTTTGC<br>AGCGACGCAAATTTTTTTTGCTGTCGTTTGGTTTTTAGTGGGGTTCTCTGTGCAACCCCACTA<br>GCTCCCTGCTTTTTCCTGCTTCACCTTCACTTCCTCGTCATCATTCAACGTGCTCTGCGTCTTTG<br>GTCATTCAGCGACGCTAACCACTTTTCCATCAATAGGAAGCCGCCGAACTCGGCAAGGGTTC<br>CTTCAAGTACGCTTGGGTTCTTGACAAGCTCAAGGCCGAGCGTGAGCGTGGTATCACCATTG<br>ACATTGCTCTGTGGAAGTTCGAGACTCCCAAGTACTATGTCACCGTCATTGGTAAGTCTTCACT<br>AAGTTCATGCTGCAATTGCGGACCAGTGCTAACAGGCAATTCACAGACGCTCCCGGCCACCG<br>TGATT
614D1	CTCGGCGGGATCTCTGCCCCGGGTGCGTCGCAGCCCCGGACCAAGGCGCCCGCCGGAGGAC<br>CAACCAAAACTCTTATTGTATACCCCCTCGCGGGTTTTTTTATAATCTGAGCCTTCTCGGCGCCT<br>CTCGTAGGCGTTTCGAAAATGAATCAAAACTTTCAACAACGGATCTCTTGGTTCTGGCATCGA<br>TGAAGAACGCAGCGAAATGCGATAAGTAATGTGAATTGCAGAATTCAGTGAATCATCGAATCT<br>TTGAACGCACATTGCGCCCGCCAGTATTCTGGCGGGCATGCCTGTCCGAGCGTCATTTCAACC<br>CTCGAACCCCTCCGGGGGGTCGGCGTTGGGGATCGGCCCTGCCTTGGCGGTGGCCGTCTCC<br>GAAATACAGTGGCGGTCTCGCCGCAGCCTCTCCTGCGCAGTAGTTTGCACACTCGCATCGGG<br>AGCGCGGCGCGTCCACAGCCGTTAAACACCCAACTTCTGAAATGTTGACCTCGGATCAGGTA<br>GGAATACCCGCTGAACTTAA	GATTCTCAAACACTTGTGCTAACCATTGCCTTCTAGGGGTGCGTATTCCATCAATCATCTTGAAT<br>GAGATCGATCGAACACAATACTGACTTGCTATAACAGCCACGTCGACTCCGGAAAGTCGACC<br>ACCGTAAGTTGCGCCCTCTCTTGCTCCGGTATCAAACGTCGTTTGATGCGGGACACCTATTCTT<br>GAACACAGGGCTAACCATGCATAATACAGACCGGTCACTTGATCTACCAGTGCGGTGGTATCG<br>ACCGTCGTACCATCGAGAAGTTCGAGAAGGTAAGCTTCAACTGATTTTCGCCTCGATTCTTCC<br>TCCTCCACATTCAATTGTGCCCGACAATTCTGCAGAGAATTTTCGTGTCGACAATTTTTCATCA<br>CCCCGCTTTCCATTACCCCTCCTTTGCAGCGACGCAAATTTTTTTTGCTGTCGTTTGGTTTTTA<br>GTGGGGTTCTCTGTGCAACCCCACTAGCTTCCTGCTTTTTCCTGCTTCACTCTCACTTCCTCGT<br>CATCATTCAACGTGCTCTGCGTCTTTGGTCATTCAGCGACGCTAACCACTTTTCCATCAATAGG<br>AAGCCGCCGAACTCGGCAAGGGTTCCTTCAAGTACGCTTGGGTTCTTGACAAGCTCAAGGC<br>CGAGCGTGAGCGTGGTATCACCATTGACATTGCTCTGTGGAAGTTCGAGACTCCCAAGTACTA<br>TGTCACCGTCATTGGTAAGTCTTCACTAAGTTCATGCTGCAATTGCGGACCAGTGCTAACAGG<br>CAATTCACAGACGCTCCCGGCCACCGTGAT
623H1	TGTGAACGTTACCAAACTGTTGCCTCGGCGGGATCTCTGCCCCGGGTGCGTCGCAGCCCCGG<br>ACCAAGGCGCCCGCCGGAGGACCAACCAAAACTCTTTTTGTATACCCCCTCGCGGGTTTTTTA<br>TAATCTGAGCCTTCTCGGCGCCTCTCGTAGGCGTTTCGAAAATGAATCAAAACTTTCAACAAC<br>GGATCTCTTGGTTCTGGCATCGATGAAGAACGCAGCGAAATGCGATAAGTAATGTGAATTGCA	CTCACGTCGGCATCATTCGCCGGTCTGATTCTCAAACTTATGCTAACCATCGCCTTCTAGGGGT<br>GCGTATTCCATCAATCATCTTGAATGAGATCGATCGAACACAATACTGACTTGCTACAACAGCC<br>ACGTCGACTCCGGAAAGTCGACCACCGTAAGTTACACCCTTTCTTGCTCCGATATCAAACGTC<br>GTTTGATGCGGGACATCTACTCTTGAACACAGGGCTAACCATTCATCATACAGACCGGTCACT

	GAATTCAGTGAATCATCGAATCTTTGAACGCACATTGCGCCCGCCAGTATTCTGGCGGGCATG<br>CCTGTCCGAGCGTCATTTCAACCCTCGAACCCCTCCGGGGGGTCGGCGTTGGGGATCGGCCC<br>TGCCTCTTGGCGGTGGCCGTCTCCGAAATACAGTGGCGGTCTCGCCGCAGCCTCTCCTGCGC<br>AGTAGTTTGCACACTCGCATCGGGAGCGCGGCGCGTCCACAGCCGTTAAACACCCAACTTCT<br>GAAATGTTGACCTCGGATCAGGTAGGAATACCCGCTGAACTTAAGCATATCAATAAGCGGAGG<br>A	TGATCTACCAGTGCGGTGGTATCGACCGTCGTACCATCGAGAAGTTCGAGAAGGTAAGCTTC<br>AACTGATTTTCGCCTCGATTCCTCTTCTTTCATATTCAATTGTGCCCGACAATTCAGAGAATTTT<br>CGTGTCGACAATTTTCATCACCCCGCTTTCCATTACCCCTCCTTTGCAGCGACGCAAATTTTTT<br>TTGCTGCCGTTTGAGTTTTAGTGGGGTTCTCTGTGCAACCCCACTAGCTCACTGCTTTTTTTGT<br>GCTTCACACTCACTTCCAAGTCATCATTCAACGTGCTCTGTGTCTTTGGTCATTCAACGATGCT<br>AACCACTTTTCCATCAATAGGAAGCCGCCGAACTCGGCAAGGGTTCCTTCAAGTACGCTTGG<br>GTTCTTGACAAGCTCAAGGCCGAGCGTGAGCGTGGTATCACCATCGACATTGCTCTGTGGAA<br>GTTCGAGACTCCCAAGTACTATGTCACCGTCATTGGTATGTCTACTTCATCAACTTCATGCTGCA<br>ATTGCAACCCAGTGCTAACAGGCAATTCAC
