# Supplementary material for: Screening of Antagonistic Trichoderma Strains to Enhance Soybean Growth
Source: J Fungi (Basel). 2025 Feb 19;11(2):159. doi: 10.3390/jof11020159 (PMC11856567; doi:10.3390/jof11020159)
Supplement: Supplementary file 1 [file jof-11-00159-s001.zip › Supplementary Table S2.pdf]

Supplementary Table S2. Comparative analysis of species diversity and community coverage across different groups.

Group	Observed species	Chao	Ace	Shannon	Simpson	Coverage
Control	535	552.158	606.625	2.234	0.084	0.998
Control	355	535.232	610.106	2.460	0.165	0.998
Control	310	448.654	602.328	2.277	0.173	0.997
223H16	460	597.962	631.153	2.419	0.209	0.997
223H16	592	745.138	735.716	3.020	0.146	0.997
223H16	412	571.642	724.806	2.884	0.119	0.997
452B7	607	699.967	709.124	2.842	0.219	0.997
452B7	598	707.837	712.315	3.665	0.089	0.997
452B7	452	616.022	675.023	3.368	0.102	0.998
561A7	439	607.098	744.546	2.452	0.203	0.997
561A7	492	720.25	826.349	3.046	0.131	0.997
561A7	411	549.392	638.396	3.034	0.111	0.997
611A17	433	645.105	753.761	3.057	0.113	0.996
611A17	562	724.358	705.444	3.283	0.091	0.997
611A17	609	748.234	725.903	4.093	0.037	0.997
625J11	484	660.949	645.592	3.499	0.06	0.997
625J11	466	647.687	766.324	3.164	0.099	0.997
625J11	494.0	741.672	852.943	3.376	0.068	0.997
