# Supplementary figures and images for: Screening of Antagonistic Trichoderma Strains to Enhance Soybean Growth
Source: J Fungi (Basel). 2025 Feb 19;11(2):159. doi: 10.3390/jof11020159 (PMC11856567; doi:10.3390/jof11020159)

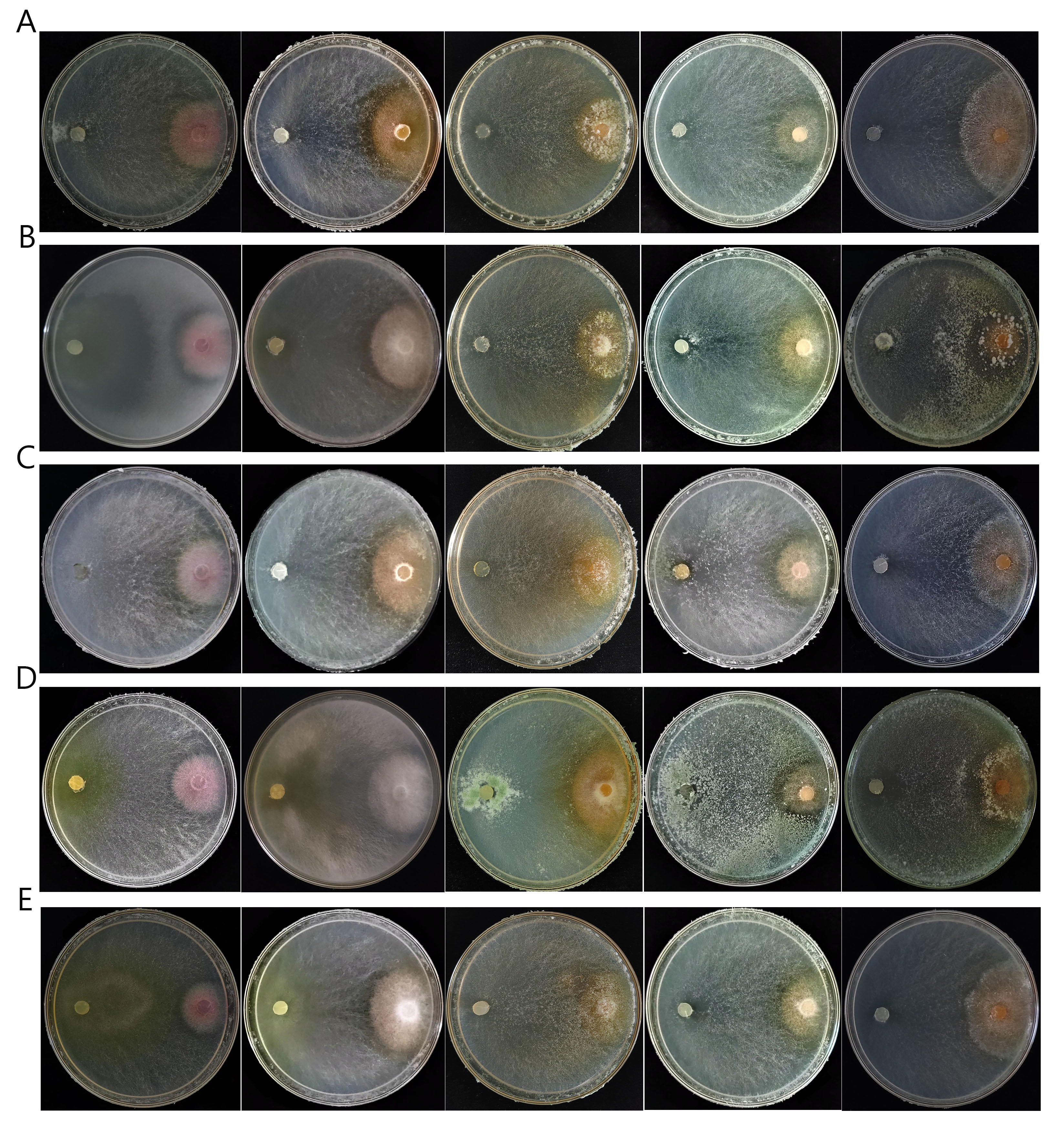

Supplement: Supplementary file 1 [file jof-11-00159-s001.zip › Supplementary Figure S1.jpg]

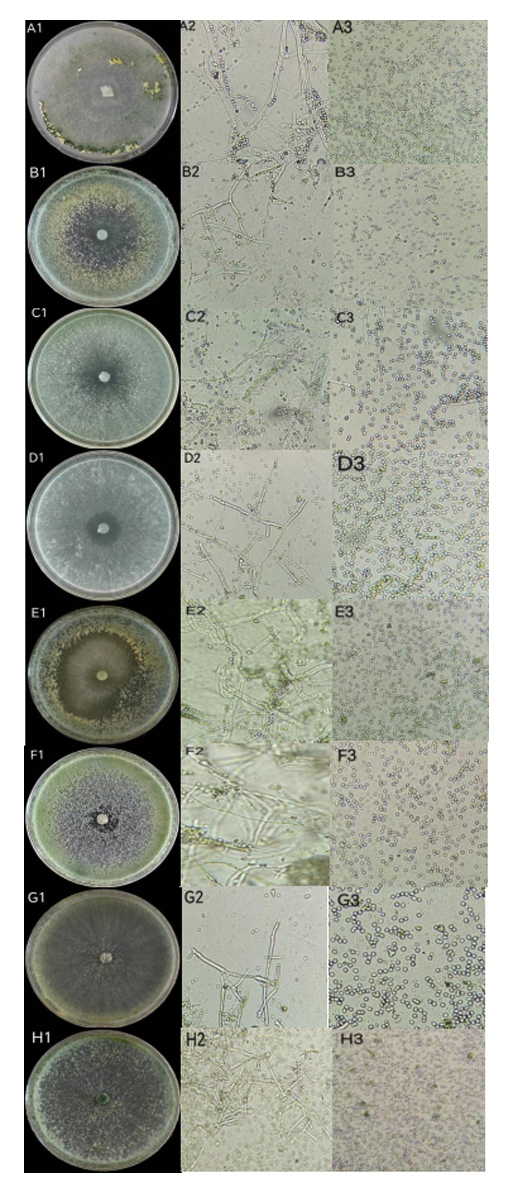

Supplement: Supplementary file 1 [file jof-11-00159-s001.zip › Supplementary Figure S2.jpg]
